# Transaldolase 1 contributes to pentose phosphate pathway disruption and synaptic dysfunction in Alzheimer’s disease

**DOI:** 10.1186/s40035-026-00567-z

**Published:** 2026-07-29

**Authors:** Xiaoyu Hu, Ying Yu, Haorui Luo, Jiabing Li, Xiaofei Zhang, Gang Wang, Jianping Li, Juan Li, Hongzhuan Chen, Yu Qiu

**Affiliations:** 1 Department of Pharmacology and Chemical Biology, Shanghai Jiao Tong University School of Medicine, Shanghai, 200025 China; 2 State Key Laboratory of Cell Biology, CAS Center for Excellence in Molecular Cell Science, Shanghai Institute of Biochemistry and Cell Biology, University of Chinese Academy of Sciences, Chinese Academy of Sciences, Shanghai, 200031 China; 3 Guangdong Provincial Key Laboratory of Stem Cell and Regenerative Medicine, Guangdong-Hong Kong Joint Laboratory for Stem Cell and Regenerative Medicine, Center for Cell Lineage Atlas, Guangzhou Institutes of Biomedicine and Health, Chinese Academy of Sciences, Guangzhou, 510530 China; 4 Department of Neurology & Institute of Neurology, Renji Hospital Affiliated to Shanghai Jiao Tong University School of Medicine, Shanghai, 200125 China; 5 Lab for Future Health, Shanghai Frotiers Science Center of TCM Chemical Biology, Shuguang Hospital, Shanghai University of Traditional Chinese Medicine, Shanghai, 201210 China

**Keywords:** Transaldolase 1, Pentose phosphate pathway, Glucose metabolism, Alzheimer’s disease, Neuron, Metabolic homeostasis, Cognition

## Abstract

**Background:**

Alzheimer’s disease (AD) is a progressive neurodegenerative disorder characterized by cognitive decline and synaptic dysfunction. Increasing evidence suggests that impaired glucose utilization is a major contributor to AD pathogenesis. Neurons preferentially use glucose through the pentose phosphate pathway (PPP). In AD, the flux through the PPP is significantly reduced; however, the underlying mechanism is still elusive. This study was aimed to elucidate how PPP was affected in AD and its contribution to the AD pathogenesis.

**Methods:**

Proteomic analyses of temporal cortex synaptosomes from AD patients and controls were conducted to identify dysregulated pathways and significantly affected proteins. Functional analysis was performed by knockdown or restoration of protein expression in primary cultured neurons, as well as in wild-type and 5 × FAD mice. Pseudotargeted metabolomics and biochemical, molecular, electrophysiological and behavioral assessments were performed to evaluate metabolic characteristics, redox status, mitochondrial function, synaptic plasticity and cognition.

**Results:**

Proteomic analysis of synaptic compartments identified glucose metabolism as the most significantly dysregulated functional network in AD. Further, transaldolase 1 (TALDO1), a rate-limiting enzyme in the PPP, was identified as a key enzyme affected in AD. TALDO1 was markedly downregulated at the early stage of AD. Downregulation of TALDO1 reduced glucose metabolism by inhibiting the PPP, TCA cycle and oxidative phosphorylation, causing broad metabolic collapse. Further, downregulation of TALDO1 depleted the nicotinamide adenine dinucleotide phosphate and glutathione pools, weakening antioxidant defense, thus resulting in mitochondria impairment and reduced energy supply. These collectively drive synaptic dysfunction and cognitive decline. Conversely, restoring TALDO1 expression in 5 × FAD mice improved glucose uptake, mitigated oxidative stress, restored metabolic homeostasis, and rescued neuronal and cognitive functions.

**Conclusion:**

These findings identify TALDO1 as a key regulator of the impaired PPP in AD and may represent a promising therapeutic target for restoring neuronal metabolic homeostasis and function.

**Supplementary Information:**

The online version contains supplementary material available at 10.1186/s40035-026-00567-z.

## Background

Alzheimer’s disease (AD) is the leading cause of dementia among the aging population worldwide, characterized by cognitive decline, behavioral abnormalities, and synaptic dysfunction [1]. The neuropathological hallmarks of AD include extracellular β-amyloid (Aβ) plaques, intracellular neurofibrillary tangles, neuronal degeneration, and widespread synaptic loss [2–5]. While these hallmarks are central to its diagnosis, a growing body of evidence indicates that global cerebral glucose hypometabolism is one of the earliest and most consistent pathophysiological features, detectable decades before clinical symptom onset [6, 7]. Positron emission tomography studies consistently demonstrated reduced cerebral metabolic rate of glucose in AD patients. This metabolic deficit correlates with disease severity and cognitive decline [7, 8].

The brain is a highly energy-demanding organ, predominantly relying on glucose metabolism to sustain neuronal activity and synaptic plasticity [9]. Impaired glucose metabolism leads to insufficient energy supply for neurons. While glycolysis and mitochondrial oxidative phosphorylation have been extensively studied [10–14], the pentose phosphate pathway (PPP), a major auxiliary route of glucose catabolism, is often overlooked in the pathophysiology of AD. However, neurons preferentially utilize the PPP to maintain their functions [15]. In neurons, PFKFB3 (phosphofructokinase-2/fructose-2,6-bisphosphatase 3) is continuously degraded by proteasomes, resulting in low activity of the key glycolytic enzyme phosphofructokinase-1 (PFK1) [16, 17]. Glucose metabolism through PPP generates nicotinamide adenine dinucleotide phosphate (NADPH) and ribose-5-phosphate (R5P), which play critical roles in the redox balance and biosynthetic processes [15–17]. The PPP flux has been reported to be significantly reduced in AD model mice [18]. Enhancing the PPP flux rescues the cognitive function of AD mice [19]. Redirecting the glucose flux to PPP mitigates tauopathy [20]. In the AD brain, mitochondria are significantly affected [21, 22], which exacerbates peroxide production, thus the generation of NADPH through PPP becomes even more critical.

Glucose-6-phosphate dehydrogenase (G6PD) is the rate-limiting enzyme of the PPP’s oxidative branch. Reduced levels of G6PD have been detected in the cerebral cortex of aged mice [23] or in the hippocampus of AD patients [24]. However, some findings show an upregulation of G6PD in AD patients [25, 26]. Transketolase (TKT), another rate-limiting enzyme involved in the PPP’s non-oxidative branch, is a thiamine-dependent enzyme. Thiamine deficiency has been linked to AD [27, 28], which may compromise the activity of TKT. On the other hand, AD brains exhibit decreased glucose transporter type 3 [29–31], which may compromise glucose delivery to neurons and then affect the PPP. However, it is still elusive whether and how the PPP is directly affected in AD.

In this study, we conducted a proteomic analysis of synaptic compartments from the temporal cortex of AD patients to investigate metabolic mechanisms underlying synaptic dysfunction. We discovered that transaldolase 1 (TALDO1) was markedly downregulated in the early-stage of AD. TALDO1, similar to TKT, is also a rate-limiting enzyme in the non-oxidative branch of the PPP. TALDO1 deficiency reduces NAPDH and glutathione (GSH) generation, leading to increased reactive oxygen species (ROS) and lipid peroxides, thereby causing mitochondrial dysfunction [32–35]. Given the essential role of the PPP in neuronal redox and energy homeostasis, we sought to determine the contribution of TALDO1-mediated metabolic regulation to AD pathogenesis and to explore whether targeting TALDO1-associated pathways may represent a potential therapeutic approach for AD.

## Materials and methods

### Human brain samples

Postmortem samples of the temporal cortex from AD patients and non-dementia controls (NDC) were sourced from the Netherlands Brain Bank at the Amsterdam Netherlands Institute for Neuroscience (open access: www.brainbank.nl). The Netherlands Brain Bank has obtained written informed consent for brain autopsy and the use of materials and clinical information for research purposes. Our study was approved by the Ethics Committee of Ruijin Hospital affiliated to Shanghai Jiao Tong University School of Medicine (2017–77). Detailed information of brain donors is provided in Table S1.

### Mice and ethics statement

All experimental procedures were approved by the Institutional Animal Care and Use Committee of Shanghai Jiao Tong University School of Medicine (A-2022–043) and conducted in accordance with their guidelines. The wild-type (WT) C57BL/6 J male mice and pregnant female mice were purchased from SLAC Laboratory Animal Co. (Shanghai, China). 5 × FAD male mice were from Jackson Laboratory (#034848, Bar Harbor, ME), and its WT male littermates were bred and randomly allocated to different groups. The mice were housed in the core animal facility of Shanghai Jiao Tong University School of Medicine. Mice were maintained in a 12-h light/dark cycle under specific pathogen-free conditions with *ad libitum* access to standard rodent chow and water. Each cage housed a maximum of five mice. Ambient temperature in the facility was maintained at 20–25 °C with a humidity of 40%–60%. Before behavioral tests, mice were transferred to the testing room and acclimated for at least 24 h to minimize stress and ensure consistent results.

### Synaptosomal crude extract

Neuronal synaptosomal crude extracts were prepared as previously described [36]. Briefly, 10 mg of human brain temporal cortex tissues or mouse hippocampal or cortex tissues were homogenized in 100 μL of pre-cooled homogenization buffer, containing 0.32 M sucrose, 4 mM HEPES, 2 mM EDTA, 50 mM NaF, 1 mM sodium orthovanadate, 0.1 mg/mL benzamidine, and a protease inhibitor cocktail (#K1008, APExBIO Technology, Shanghai, China), pH 7.4. The tissue homogenate was centrifuged at 1000 × *g* for 10 min at 4 °C to obtain supernatant S1. The S1 was then centrifuged at 10,000 × *g* for 15 min at 4 °C, and the supernatant was discarded. The pellet was resuspended in 0.32 M sucrose-HEPES buffer (volume ratio 1:10) and recentrifuged at 10,000 × *g* for 15 min at 4 °C. The resulting pellet, representing the crude synaptosomal fraction (P2), was then used for proteomic analysis, or for Western blotting after lysis and denaturation.

### Label-free quantitative proteomic analysis

The synaptosomal crude extracts (P2 fraction) from human brain temporal cortex samples were analyzed by non-targeted quantitative proteomics in the following steps. Protein digestion: P2 pellets were lysed with 8 M urea in 0.1 M pH 8.5 Tris buffer containing 1:5000 dilution of Universal nuclease (#88700, Thermo Fisher Scientific, Waltham, MA) using a dounce homogenizer. The soluble synaptosomal lysates were collected after maximum speed centrifugation at room temperature for 15 min. Whole proteome peptides were prepared using the Filter Aided Sample Preparation protocol as described before [37]. Briefly, 50 μg of protein was loaded onto a 30 kDa filter and centrifuged at 12,000 × *g* for 15 min at 20 °C. Alkylation was performed with 50 mM iodoacetamide in urea buffer for 15 min at 20 °C. After washing, proteins were digested overnight at 37 °C with 500 ng of trypsin in 50 mM ammonium bicarbonate buffer. Peptides were extracted and acidified with trifluoroacetic acid. Stage tipping was performed as described before [38]. Mass spectrometry: peptides were separated using an Easy-nLC 1200 system with a 240-min data collection (11 min of 2%–7%, 200 min 7%–28%, 15 min of 28%–36% and 5 min of 36%–60% gradient of buffer B) for peptide separation, followed by two washes: 2 min of 60%–95% and 7 min of 95% buffer B, connected online to a Fusion Lumos mass spectrometer with FAIMS pro (Thermo Fisher Scientific). Scans were collected in a data-dependent top-speed mode with dynamic exclusion at 90 s. Raw data were analyzed using the MaxQuant version 1.6.0.1 search against human Fasta database using default settings [39], with label-free quantification and match between runs functions enabled. The output protein list was analyzed and visualized using DEP package as described previously [40].

Data analysis: after standardizing the data, principal component analysis (PCA) was performed using the prcomp function in R, and volcano plots were generated with the ggplot2 package. Differentially expressed protein-encoding genes were uploaded to the DAVID database (https://david.ncifcrf.gov/) and Metascape (https://metascape.org/) for pathway enrichment analysis. For Kyoto Encyclopedia of Genes and Genomes (KEGG) pathway analysis, enriched pathways were ranked by a composite score integrating enrichment significance, the number of hit proteins (count), and the expression changes of the corresponding proteins. The top 30 pathways were selected for visualization. Among the proteins involved in these pathways, 15 proteins with the most pronounced expression changes were highlighted and visualized using chord plots generated in R. For Reactome pathway analysis, pathways were ranked using the same criteria, including enrichment significance, hit protein counts, and expression changes. The top 30 enriched pathways were selected, and chord plots were generated in R to display these pathways together with the 15 proteins showing the most prominent expression changes. Gene Set Enrichment Analysis (GSEA) was performed using the GSEA software (http://www.gsea-msigdb.org/). The analysis was performed using the ranked list of all quantified proteins from the entire proteomic expression profile. The group information files were formatted in accordance with the specifications outlined on the official GSEA website. Subsequently, these files were uploaded and analyzed utilizing established gene sets from the official database to identify enriched pathways and genes. Protein interactions were analyzed using the STRING website (https://cn.string-db.org/), and protein interaction network maps were produced using Cytoscape software.

### Pseudotargeted metabolomics

For pseudotargeted metabolomics, cells were harvested in six-well plates, washed by cold PBS buffer twice and immediately quenched in liquid nitrogen. All samples were lysed in 1 mL of − 80 °C extraction solvent (80% methanol/water). After centrifugation (20,000 × *g*, 4 °C, 15 min), the supernatant was transferred to a new tube, and samples were dried using a vacuum centrifugal concentrator. Metabolites were reconstituted in 200 μL of 80% acetonitrile/water, vortexed, and centrifuged to remove insoluble material. All samples were stored at − 80 °C before LC–MS/MS analysis.

Metabolites with *P* < 0.05, and fold change (sh*Taldo1*/shCtl) > 1.2 or < 0.8 were considered as differential metabolites. PCA was performed using the prcomp function in R, and volcano plots were generated with the ggplot2 package.

### Preparation of Aβ_42_ and oligomers

Briefly, 1 mg of Aβ_42_ (#NBP3-18318, Novus Biologicals, Centennial, CO) was dissolved in 1 mL of HFIP (#H0424, Tokyo Chemical Industry Co., Ltd., Tokyo, Japan) on ice. After 10-min incubation, the solution was aliquoted into centrifuge tubes and concentrated via vacuum centrifugation for 15 min to fully evaporate HFIP. The residue was then solubilized in DMSO, sonicated at 75% power for 10 min, and diluted in PBS to yield a 5 mM Aβ_42_ monomer stock solution. To prepare Aβ_42_ oligomers, the 5 mM Aβ_42_ monomer stock solution was diluted to 100 μM in sterile PBS in triple-distilled water, and incubated at 4 °C for 24 h to promote oligomerization.

### Stable *Taldo1* knockdown in HT22 cells

Two shRNAs targeting mouse *Taldo1* (shT1 targeting sequence CCTTTGAGCTGGGTCCTAATT or shT2 targeting sequence GAAGATTCCAGGCCGTGTATC) were respectively inserted into the pSIREN RetroQ vector (#631526, Clontech Laboratories, Mountain View, CA). HEK293T cells (ATCC, CRL-3216, Manassas, VA) were transfected with the retroviral expression constructs and the helper plasmid pHelper using Lipofectamine 3000 (Thermo Fisher Scientific). After 72 h, supernatant was collected, centrifuged to remove cellular debris, and filtered through a 0.45-μm membrane to obtain the recombinant viruses. HT22 cells (#CL-0697, Procell Life Science & Technology Co., Ltd., Wuhan, China) were then infected with the recombinant viruses and subjected to puromycin selection to obtain stable shRNA-expressing pooled clones.

### Viruses

LV-U6-sh*Taldo1* (shT1)-CMV-mcherry-F2A-Puro-WPRE and its corresponding control (shCtl) (Obio Tech, Shanghai, China) were used for infection of primary cultured neurons. pAAV-hSyn-mCherry-shT1-mir30 arm and its shCtl (titer: 1 × 10^13^ viral genomes per mL, Sunbio Medical Biotechnology Co., Ltd., Shanghai, China) were injected into the bilateral ventricles of mice. LV-hSyn- *Taldo1*-3Flag-2A-EGFP and its corresponding control (titer: 1 × 10^9^ viral genomes per mL, Sunbio Medical) were used for both infection of primary cultured neurons and injection into the bilateral ventricles of mice.

### Primary cortical neuron culture and infection

Primary neurons were isolated from C57BL/6 J embryos on day 16, as previously described [41]. Briefly, brain cortices were dissected from pups, with meninges removed and cortical tissue dissociated via enzymatic digestion. Isolated primary neurons were plated on poly-d-lysine-coated dishes and cultured in Neurobasal medium containing B27 (#17504044, Thermo Fisher Scientific) and 1% penicillin/streptomycin (#15140122, Thermo Fisher Scientific) in a 5% CO_2_ incubator at 37 °C. Lentivirus infection was performed on day 4. For *Taldo1* knockdown followed by overexpression, neurons were cultured with half-volume medium changes and were transduced on day 4 with control lentivirus or lentivirus expressing *Taldo1* shRNA. On day 7, neurons were transduced with control lentivirus or lentivirus expressing *Taldo1*. Synaptic analyses were performed on days 18–23, when neurons exhibit mature synaptic networks.

### Lateral ventricular virus injection

For intracerebroventricular injection of virus, mice were anesthetized by inhalation of 2% isoflurane and mounted on a stereotaxic frame. For 5-month-old C57BL/6 J mice, AAVs were injected into the bilateral ventricles at the following coordinates: anteroposterior, − 0.6 mm; lateral, ± 1.5 mm; and ventral, 2.2 mm; relative to bregma. For 3-month-old 5 × FAD and WT mice, lentiviruses were injected into the bilateral ventricles at the following coordinates: anteroposterior, − 0.3 mm; lateral, ± 1.0 mm; and ventral, 2.0 mm; relative to bregma. Mice in the control group were injected with corresponding control AAVs or LVs. 2 μL of AAVs or 3 μL of LVs were slowly injected over 10 min, followed by a 10-min pause to facilitate diffusion. The needle was then slowly withdrawn over the course of 2 min. Pathological assessments and behavioral tests were performed 2 months or 45 days after viral injection.

### Transmission electron microscopy (TEM)

Fresh synaptosomal pellets or 1-mm^3^ tissues were fixed in 2.5% glutaraldehyde at 4 °C for 24 h. The samples were then transferred to 1% osmium tetroxide solution for 1 h at room temperature for postfixation. Subsequently, the samples were dehydrated in a graded ethanol series and embedded in epoxy resin. Ultrathin sections were cut using an ultramicrotome, stained with lead citrate, and examined under a JEOL JEM-1010 transmission electron microscope operated at an acceleration voltage of 80 kV.

### NADP^+^ and NADPH assays

NADP^+^ and NADPH levels in HT22 cells, mouse primary neurons, and brain tissue were measured using the NADP(H) Assay Kit (#KTB1010, Abbkine Scientific Co., Ltd., Wuhan, China) according to the manufacturer’s instructions. For cells, about 1 × 10^6^ cells were collected, washed twice with ice-cold PBS, and centrifuged at 800 × *g* for 5 min at 4 °C. The supernatant was discarded to retain the cell pellet. For tissues, about 20 mg of tissue blocks were rinsed with ice-cold PBS, and quickly cut into small pieces with scissors. NADP⁺ and NADPH were extracted separately using 100 μL NADP⁺ extraction buffer and 100 μL NADPH extraction buffer, respectively. Extracts were heated at 60 °C for 5 min, neutralized by adding 20 μL of assay buffer and 100 μL of the opposite extraction buffer, vortexed, and centrifuged at 14,000 × *g* for 5 min. Supernatants were collected, and NADP^+^ and NADPH levels were measured according to the instructions.

### Glutathione (GSH) assay

GSH was measured using the GSH Assay Kit (#BC1175, Solarbio, Beijing, China) according to the manufacturer’s instructions. Fresh tissues were rinsed twice with PBS, and 0.1 g of tissue was homogenized in 1 mL lysis buffer on ice until no visible clumps remained. The samples were then centrifuged at 8000 × *g* for 10 min at 4 °C to collect the supernatant. For cells, at least 5 × 10^6^ cells were collected, washed twice with PBS, and centrifuged at 1000 × *g* for 5 min at 4 °C to obtain the cell pellet. The pellet was resuspended in 1 mL lysis buffer, subjected to 2–3 freeze–thaw cycles using liquid nitrogen and a 37 °C water bath, and then centrifuged at 8000 × *g* for 10 min at 4 °C to collect the supernatant. The GSH level in the supernatants was measured according to the instructions.

### Detection of mitochondrial membrane potential

Mitochondrial membrane potential was assessed using the JC-10 Mitochondrial Membrane Potential Assay Kit (#CA1310, Solarbio) according to the manufacturer’s instructions. For cell-based assays, primary neurons or HT22 cells were cultured in confocal dishes. After treatment, the culture medium was removed and replaced with JC-10 staining working solution. Cells were incubated at 37 °C for 20 min, followed by two washes with JC-10 staining buffer. Subsequently, neuronal medium or DMEM complete medium was added. The red (aggregate)/green (monomer) fluorescence intensity ratio in each group was quickly assessed under a confocal microscope to determine the degree of mitochondrial membrane potential depolarization. For tissue samples, mitochondria were isolated using the Tissue Mitochondria Isolation Kit (#C3606, Beyotime, Shanghai, China). Purified mitochondria were stained with the JC-10 working solution and incubated according to the manufacturer’s protocol. Fluorescence intensity was then measured using a microplate reader (excitation/emission wavelengths: 490/530 nm for monomer; 525/590 nm for aggregate).

### ROS detection

Cells were seeded in confocal culture dishes. When the cell confluence reached 60%–70%, CellROX® reagent (#C10422, Thermo Fisher Scientific) was added to the culture medium to a final concentration of 5 μM, and cells were incubated at 37 °C for 30 min. After removing the medium, cells were washed with pre-warmed PBS, fixed in 3.7% paraformaldehyde for 15 min at room temperature, and stained with Hoechst for nuclear visualization. Fluorescence intensity was measured, and data were collected within 2 h (excitation wavelength: 561 nm; emission wavelength: 580–590 nm).

### Oxygen consumption assessment

Cellular oxygen consumption rates (OCR) (pmol/min) was measured using the Seahorse XF24 Analyzer (Agilent, Santa Clara, CA) with a modified protocol. Briefly, freshly isolated primary neurons or HT22 cells were seeded in poly-L-lysine-coated Seahorse XF24 microplates at 80%–90% confluency. Cells were equilibrated at 37 °C in a non-CO₂ incubator for 45–60 min prior to analysis. In OCR measurements, 56 μL oligomycin (1.5 μM), 62 μL FCCP (1 μM), and 69 μL rotenone/antimycin A (0.5 μM) were sequentially added to ports A–C of the hydrated sensor cartridge. The cell plate was then transferred to the XF24 Analyzer for mitochondrial OCR detection. Data were analyzed using Seahorse Wave software.

### Immunofluorescence staining

Mice were anesthetized with sodium pentobarbital (50 mg/kg, intraperitoneal injection) and perfused with 0.9% saline and then with 4% paraformaldehyde. Brains were harvested and post-fixed in 4% paraformaldehyde overnight, transferred to 10% sucrose for three days, and then to 30% sucrose for another three days. Brain tissues were embedded in OCT and sectioned at 20 μm on a cryostat. Sections were rinsed in PBS for 10 min, blocked in PBS with 5% BSA for 1 h, and incubated with primary antibodies overnight at 4 °C. The next day, following three PBS washes, sections were incubated with secondary antibodies at room temperature for 1 h. After three additional PBS washes, sections were mounted with ProLong™ Gold Antifade Mountant with DAPI (#D3571, Life Technologies, Eugene, OR).

Cells were previously seeded in confocal culture dishes. The culture medium was discarded, and cells were rinsed three times with PBS. Cells were fixed in 4% paraformaldehyde, and then blocked with immunofluorescence blocking solution at room temperature for 30 min. The cells were incubated overnight with primary antibodies at 4 °C. After three washes with PBS, the cells were incubated with secondary antibodies in a humid chamber at room temperature for 1 h. After three PBS washes, sections were incubated with diluted DAPI for 15 min and then rinsed with PBS. Fluorescent images were acquired using a Leica SP8 confocal microscope (Leica, Wetzlar, Germany). The primary antibodies used are listed below: anti-TALDO1 (1:200, KleanAB, P101222, China), anti-NeuN (1:500, Abcam, ab104224, Cambridge, UK), anti-Iba1 (1:300, Servicebio, GB2105, Wuhan, China), anti-GFAP (1:200, Cell Signaling Technology, #3670, Danvers, MA), anti-β-Amyloid (1:200, Cell Signaling Technology, #8243), anti-Synaptophysin (1:50, Santa Cruz, sc-17750, Dallas, TX), anti-PSD95 (1:200, Abcam, ab18258), MAP2 (1:300, Cell Signaling Technology, #4542), anti-G6PD (1:150, Proteintech, 25413–1-AP, Wuhan, China), and anti-TKT (1:300, Proteintech, 11039–1-AP). The secondary antibodies used are listed below: goat anti-rabbit IgG H&L (Alexa Fluor® 488) (1:1000, Abcam, ab150077) and goat anti-mouse IgG H&L (Alexa Fluor® 555) (1:1000, Abcam, ab150114).

### Quantitative real-time PCR

Total RNA was extracted from cells using the SimplyP Total RNA Extraction Kit (Bioer, Hangzhou, China) and from mouse tissues using TRIzol Reagent (Thermo Fisher Scientific), following the manufacturers’ protocols. Extracted RNA (2 μg) was reverse-transcribed into cDNA with PrimeScript™ RT Master Mix (#RR036A,Takara, Shiga, Japan). Quantitative PCR was performed using TB Green™ Premix Ex Taq™ (#RR820A,Takara) on a LightCycler 480 System (Roche Diagnostics, Mannheim, Germany). β-Actin served as the housekeeping gene. Gene expression level was analyzed using the 2^−ΔΔCt^ method. The primers used are listed below: *Taldo1* forward: 5’- GTGGGGCGCATCCTTGATT -3’; reverse: 5’- TGGTCTTGTAGCCGAACTTCT -3’; *GFAP* forward: 5’- GCGGGATGGAGAGGTCATTA -3’; reverse: 5’- GCGGAGCAACTATCCTGCTT -3’; β-actin forward: 5’- TCCGTAAAGACCTCTATGCCAACAC -3’; reverse: 5’- GTACTCCTGCTTGCTGATCCACAT -3’.

### Western blotting

Total protein was extracted using RIPA buffer (Beyotime) containing a protease/phosphatase inhibitor cocktail (#P1045, Beyotime). Equal amounts (20 µg) of each protein sample were separated on 8%–12% gradient SDS-PAGE gels and transferred to polyvinylidene fluoride membranes. Non-specific binding was blocked with 5% non-fat milk in 1 × Tris-buffered saline for 1 h at room temperature. Membranes were incubated with primary antibodies overnight at 4 °C, followed by 1-h incubation with HRP-conjugated or fluorescent secondary antibodies at room temperature. After each antibody incubation, membranes were washed three times with TBST for 15 min each. Immunocomplex bands were visualized using an ECL kit (#P0018S, Beyotime) and analyzed with the Li-Cor Odyssey Fc Image Studio (Li-Cor Biosciences, Lincoln, NE). Immunofluorescence intensities were normalized to total protein or β-actin. For proteins with similar molecular weights, the same antibody source was used for blotting, and lysates were evenly split and subjected to SDS-PAGE separately. The following antibodies were used: anti-TALDO1 (1:1000, KleanAB, P101222), anti-Phospho-AMPKα (1:1000, Cell Signaling Technology, 50081), anti-AMPKα (1:1000, Cell Signaling Technology, 5831S), anti-Iba1 (1:1000, Servicebio, GB2105), anti-GFAP (1:1000, Cell Signaling Technology, 3670), anti-Tuj1 (1:1000, Abcam, ab18207), and anti-β-Actin (1:10,000, Proteintech, 66009–1-Ig). Secondary antibodies included Anti-rabbit IgG, HRP-linked Antibody (1:3000, Cell Signaling Technology, #7074), Anti-mouse IgG, HRP-linked Antibody (1:3000, Cell Signaling Technology, #7076), and IRDye goat anti-rabbit or -mouse IgG (H + L) (1:10,000, Li-Cor Biosciences).

### Golgi staining and spine analysis

Golgi staining was performed using the FD Rapid Golgi Stain™ Kit (#PK401, FD NeuroTechnologies, Ellicott City, MD) following the manufacturer’s instructions to detect neuronal dendritic spines. Brain sections were cut at 100-µm thickness and washed in ascending concentrations of ethanol (50%, 75%, 95%, and 100%), followed by xylene washing. Sections were then mounted with a resin-based mounting medium and air-dried away from light. Staining was observed under a Leica DFC320 microscope. Spine density was measured on distal dendrites located over 50 µm from the soma. Spine number and dendritic length were analyzed with the ImageJ software, with the total length automatically accumulated across all traced segments. Spine density was calculated as the total number of spines divided by the accumulated dendritic length (spines/10 μm).

### Nissl’s staining

Mouse brain paraffin sections were sequentially immersed in xylene, anhydrous ethanol, 90% ethanol, 75% ethanol, and distilled water for 5 min to rehydrate. Then, the sections were stained with Nissl solution containing 1% toluidine blue at 60 °C for 1 h, with hydrochloric acid added to enhance neuron staining. After staining, the sections were rinsed with distilled water to remove residual dyes and reagents, differentiated in 95% ethanol for 2 min, dehydrated with 95% ethanol and anhydrous ethanol for 5 min each, cleared with xylene twice for 5 min each, and finally mounted with a neutral resin medium. Staining was observed under a Leica DFC320 microscope.

### Positron emission tomography/computed tomography (PET/CT) scans

^18^F-fluorodeoxyglucose (^18^F-FDG) radiotracer was provided by the Small Animal PET/CT Center of Ruijin Hospital Affiliated to Shanghai Jiao Tong University School of Medicine. After 8-h fasting, each mouse was injected with 180–200 μCi of ^18^F-FDG via the tail vein followed by a 50-min distribution period. The mice were anesthetized with 2% isoflurane by inhalation using a vaporizer and then underwent head scanning using the Inveon micro-PET/CT scanner (Siemens Medical Solutions USA, Knoxville, TN).

### Behavioral tests

Behavioral assessment involving male C57 mice with *Taldo1* knockdown was performed at the age of five months, and that involving male 5 × FAD and WT mice with *Taldo1* overexpression was at the age of three months. Behavioral experiments were performed 1.5–2 months after virus injection.

#### Open field test

The open field test was employed to assess locomotor activity and anxiety-like behavior in mice. Each mouse was gently placed in the center of a square arena (30 cm × 30 cm × 30 cm) designed for open field testing. The environment was uniformly illuminated to ensure consistent visibility, and the floor was marked with a grid to facilitate subsequent analysis of movement patterns. During the test, each mouse was allowed to explore the environment freely for a 5 min, during which their movements were continuously recorded by a video tracking system (Mobile Datum, Shanghai, China). This system enabled precise quantification of various parameters, including total distance traveled, velocity, time spent in the center versus peripheral zones, and patterns of exploration. The data obtained provided insights into both locomotor function and anxiety-related behavior, with reduced central exploration typically indicating higher anxiety levels.

#### Novel object recognition (NOR)

NOR test was used to assess non-spatial memory in mice, with a modified procedure [42]. On day 1, mice were acclimated to an open field arena (30 cm × 30 cm × 30 cm) for 5 min. On day 2, they were placed in the arena with two identical objects for 5 min (training). In the test trial 24 h later, mice were returned to the arena for 5 min with one familiar and one novel object. The discrimination index was calculated as (time exploring familiar object − time exploring new object) / total exploration time. This index provides a quantitative assessment of non-spatial memory, with higher values indicating better recognition memory.

#### Y maze

The Y-maze spontaneous alternation test was used to evaluate spatial working memory. The test was performed in an opaque Perspex Y-maze with three arms measuring 30 cm long, 8 cm in wide, and 15 cm high, with 120° angles between each two arms. Each mouse was placed on the edge of arm A and allowed to explore the maze freely for 5 min. The entries into each arm were recorded. Spontaneous alternation was defined as sequential entries into three distinct arms. The alternation rate was calculated as follows: (number of effective alternations / total number of entries) × 100%.

#### Morris water maze (MWM)

MWM test was performed according to the previously described protocol with minor modifications [36, 43]. Mice were trained in a circular pool (150-cm diameter) containing opaque water (30 cm deep, 19–22 °C). A white platform (6-cm diameter) was positioned 1 cm below the water surface in the center of a designated quadrant. Over five days of training, mice underwent four daily trials in different quadrants to locate the hidden platform. Mice that failed to find the platform within 60 s were guided to the platform and stayed there for 30 s. On the final day, a probe trial was conducted with the platform removed, during which each mouse was allowed to swim for 30 s. Swimming paths and escape latencies were recorded and analyzed using a video tracking system (Mobile Datum).

### Field excitatory postsynaptic potential (fEPSPs) slope measurement

The recording of fEPSPs followed a previously reported method [44] with minor modifications. Mice were anesthetized via intraperitoneal injection of sodium pentobarbital at 40 mg/kg before decapitation. Hippocampal slices (370 μm) were prepared using a Vibratome (Vibratome 3000, Vibratome Company, St. Louis, MO) in modified artificial cerebrospinal fluid (aCSF) containing 2.5 mM KCl, 0.5 mM CaCl_2_, 7 mM MgSO_4_, 25 mM NaHCO_3_, 1.25 mM NaH_2_PO_4_, 25 mM D-glucose, and 110 mM choline chloride, pH 7.4, bubbled with 95% O_2_ and 5% CO_2_ at 4 °C. The slices were then transferred to a chamber with normal aCSF containing 119 mM NaCl, 2.5 mM CaCl_2_, 2.5 mM KCl, 1.3 mM MgSO_4_, 26.2 mM NaHCO_3_, 1.0 mM Na_2_HPO_4_, and 11 mM D-glucose, pH 7.4, oxygenated with 95% O_2_ and 5% CO_2_, for 60 min at 31 °C. For stimulation, a monopolar tungsten electrode (FHC, Bowdoin, ME) was placed in the Schaffer collateral fibers to deliver electrical pulses. Extracellular field potentials were recorded using glass microelectrodes (3 MΩ, 0.1 M CH_3_COONa) positioned in the CA1 stratum radiatum. The test pulses were applied at 0.033 Hz, with each stimulus pulse lasting 0.01 ms and having an intensity of 0.03–0.1 mA. Data acquisition involved filtering (high-pass 0.1 Hz, low-pass 3 kHz), followed by amplification and digitization using an Axoclamp 2B amplifier and Digidata 1322 interface (Axon Instruments/Molecular Devices, Union City, CA). For long-term potentiation (LTP) recording, baseline fEPSPs were monitored for at least 15 min at 1/3 intensity of that required for maximal fEPSP evocation. LTP was induced via three trains of theta burst stimulation, each comprising 10 bursts of four pulses delivered at 100 Hz with 200 ms intervals between bursts. Following theta burst stimulation, fEPSP recording was continued for an additional 45 min.

### Enzyme-linked immunosorbent assay (ELISA)

Aβ_40_ and Aβ_42_ levels were quantified using a commercial ELISA kit (#KHB3481, #KHB3441, Thermo Fisher Scientific) following the manufacturer’s instructions. Soluble proteins were obtained from mouse cortex and hippocampus by homogenization in PBS. For insoluble Aβ extraction, guanidine hydrochloride (6.25 M), 50 mM Tris, and 1% PMSF (pH 8.0) were added to the precipitate from PBS homogenization. This mixture was sonicated for 2 h and then centrifuged at 16,000 × *g* for 1 h at 4 °C. The supernatant containing insoluble Aβ proteins was collected after centrifugation. Absorbance was measured at 450 nm using a Multifunctional Enzyme Labeler (Thermo Fisher Scientific). TALDO1 levels were measured using a commercial kit (#SEB371Mu, Cloud-Clone, Houston, TX) following the manufacturer’s instructions. The procedure included antibody incubation, washing, HRP conjugation, another wash, substrate reaction, reaction termination, and finally, absorbance reading.

### Statistics

All data are presented as means ± standard error of the mean (SEM). Sample sizes (*n*) in the figure legends represent the number of independent experiments for cellular experiments or the number of mice for experiments involving mice or tissues from mice, unless otherwise specified. Statistical analyses were conducted using GraphPad Prism 9.0. Comparisons between two groups were performed using unpaired two-tailed Student’s *t*-test. For comparisons involving three or more groups, one-way, two-way, or three-way ANOVA was employed, followed by appropriate post-hoc analyses. Data that were not normally distributed were analyzed with the non-parametric two-tailed Mann–Whitney test between two groups. Pearson correlation analysis was utilized to evaluate relationships between two factors. *P* < 0.05 was considered statistically significant.

## Results

### Proteomic analysis of neuronal synaptosomes identifies glucose metabolism as a key pathway affected in AD

**Fig. 1:**
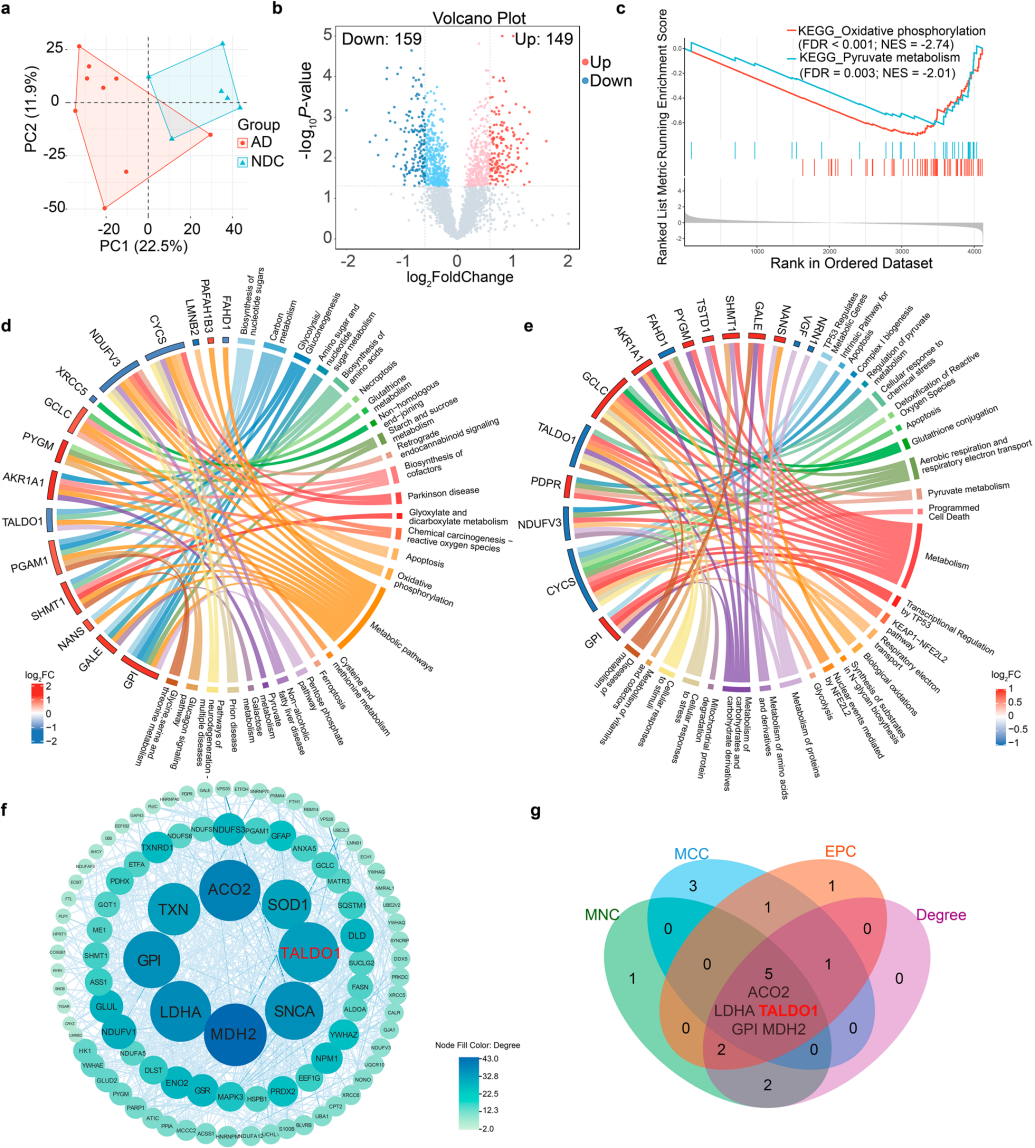
Proteomics of synaptosomes in the temporal cortex of AD patients. **a** PCA of proteomic data from AD patients (*n* = 9) and non-dementia controls (NDC) (*n* = 6). **b** Volcano plot indicates the differentially expressed proteins of AD versus NDC, with red representing upregulated proteins in AD and blue representing downregulated proteins in AD (*P* < 0.05, log_2  _fold change higher than 0.66 or lower than −0.66). Proteins with missing values greater than 1 were excluded from the analysis. The X-axis represents log_2_ of the fold change between AD and NDC and the Y-axis shows the negative log_10_ of *P* value. **c** GSEA analysis of the ranked list of all quantified proteins revealed enrichment of the most significantly downregulated oxidative phosphorylation pathway and pyruvate metabolism pathway. **d**, **e** KEGG pathway enrichment (**d**) and Reactome pathway enrichment (**e**) analysis of differentially expressed proteins. Proteins are colored according to log_2_ fold change (red, upregulated; blue, downregulated). **f** Differential protein interaction network map was generated using Cytoscape software. **g** Venn diagram analysis of key node proteins identified by four topological algorithms (MNC, MCC, EPC, and Degree) based on the PPI network. The overlapping region represents five high-confidence hub proteins (ACO2, LDHA, GPI, MDH2, and TALDO1)

Abnormal neuronal degeneration and synaptic dysfunction are critical features of the pathogenesis of AD. To gain insights into the altered protein expression in AD neurons and synapses, we examined the proteome abundance of crude synaptosomal fractions from the temporal cortex of AD patients and NDC using label-free proteomics (Fig. S1a). TEM revealed clear and intact synaptic structures, with the presynaptic membrane containing synaptic vesicles and the postsynaptic membrane exhibiting dense material (Fig. S1b), demonstrating the integrity of our synaptosomal preparation. PCA illustrated differences between AD and NDC samples (Fig. 1a). Comprehensive analyses of the enriched peptides identified 149 upregulated and 159 downregulated proteins in AD compared to NDC (Fig. 1b, Table S2).

GSEA analysis of the ranked list of all quantified proteins revealed enrichment of glucose and energy metabolism pathways, with oxidative phosphorylation and pyruvate metabolism ranking as the most significantly downregulated pathways (Fig. 1c). KEGG pathway enrichment analysis of the differentially expressed proteins further highlighted metabolic dysregulation, particularly dysregulation of carbon metabolism, glycolysis/gluconeogenesis, pyruvate metabolism, and PPP (Fig. 1d). In addition, several pathways associated with neurodegenerative diseases, including Parkinson disease, prion disease, and pathways of neurodegeneration—multiple diseases, were also enriched, suggesting potential links between metabolic dysfunction and neuronal impairment. Consistently, reactome pathway analysis revealed significant enrichment of pathways related to mitochondrial respiration, oxidative stress responses, and metabolic regulation, including respiratory electron transport, glutathione conjugation, and KEAP1 (Kelch-like ECH-associated protein 1)–NFE2L2 (Nuclear factor erythroid-derived 2-like 2) pathway (Fig. 1e). Together, these analyses consistently indicate that the neuronal metabolic pathways—particularly those involved in glucose utilization, mitochondrial respiration, and redox homeostasis—are profoundly disrupted in AD. Therefore, metabolic dysfunction may represent a central mechanism contributing to neuronal impairment.

Furthermore, protein–protein interaction network analysis using STRING (https://string-db.org/), visualized in Cytoscape, identified seven proteins forming the core metabolic regulatory module of the network, including MDH2, ACO2, TALDO1, SOD1, LDHA, SNCA, and TXN (Fig. 1f). Topological analysis using multiple centrality algorithms (MNC, MCC, EPC, and Degree) prioritized five high-confidence nodes: ACO2, LDHA, GPI, MDH2, and TALDO1 (Fig. 1g). Among these proteins, ACO2, LDHA, GPI, and MDH2 have been reported to be related with metabolic dysfunctions in AD [45–49]. TALDO1, which was downregulated, and the only novel, uncharacterized component within the network, is a key enzyme of the PPP, intriguing us to conduct an in-depth investigation.

### TALDO1 is reduced in AD

**Fig. 2:**
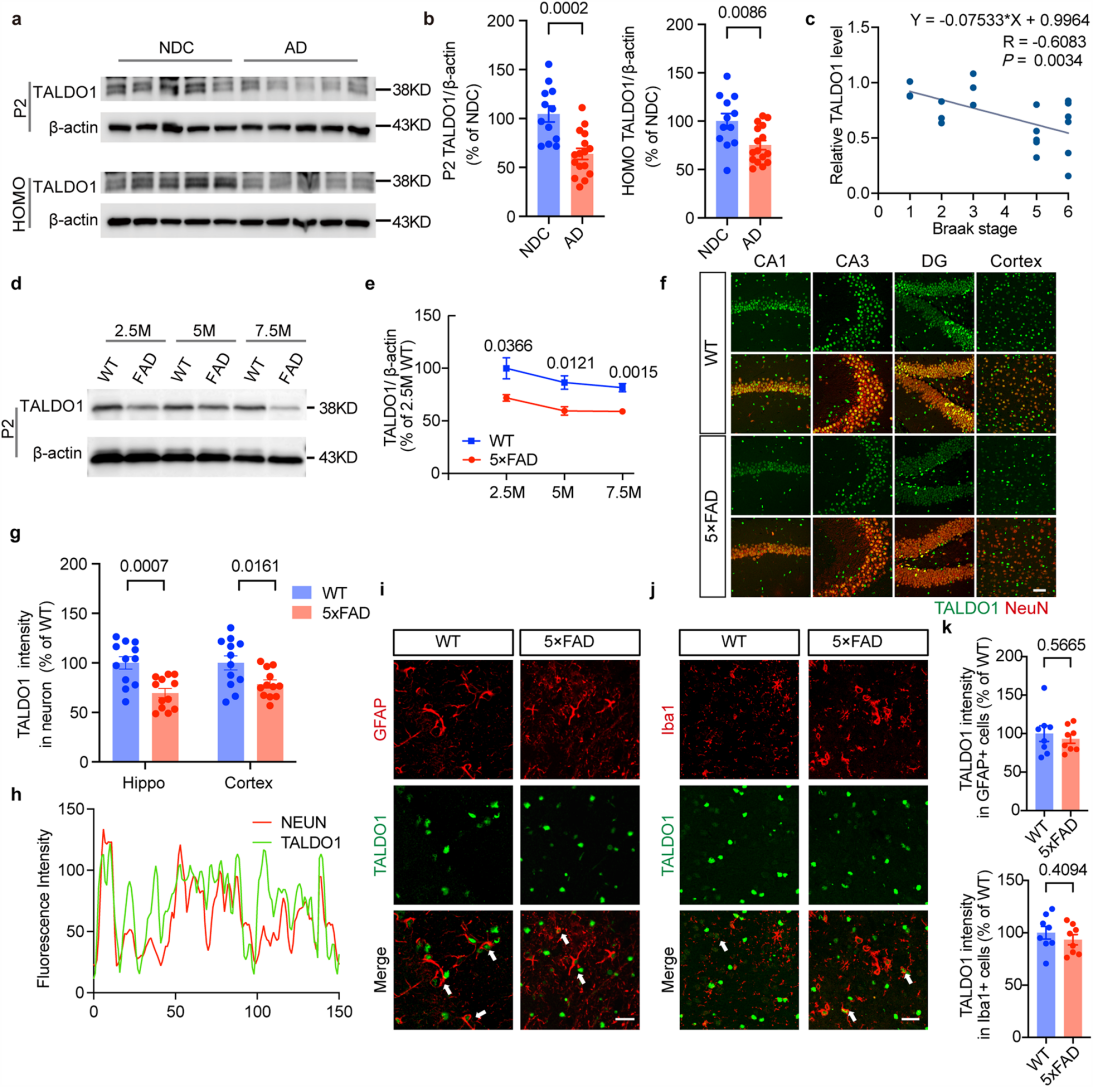
TALDO1 is downregulated in neurons of AD brains. **a**, **b** Western blot analysis (**a**) and quantification of TALDO1 (**b**) in synaptosomal crude extracts and homogenates from the temporal cortex of AD patients (*n* = 16) compared to NDC (*n* = 12). P2: synaptosomal crude extracts, HOMO: homogenates. **c** The correlation between TALDO1 expression level in the homogenates and Braak stage was assessed using linear regression (*n* = 11 for NDC and *n* =10 for AD). **d, e** Western blot analysis (**d**) and quantification (**e**) of TALDO1 in synaptosomal crude extracts from 2.5, 5, and 7.5-month-old WT and 5 × FAD mice (*n* = 4). P2: synaptosomal crude extracts.** f**, **g** Confocal images (**f**) and quantification (**g**) of TALDO1 in neurons in the hippocampal CA3, CA1 and DG regions, and cortex of 2.5-month-old WT and 5 × FAD mice (*n* = 12 slices from 4 mice per group). Scale bar, 40 μm. **h** Co-localization of fluorescence between TALDO1 and NeuN. **i**–**k** Confocal images (**i**,** j**) and quantification (**k**) of TALDO1 in astrocytes and microglia of 2.5-month-old WT and 5 × FAD mice (*n* = 8 slices from 4 mice per group). Scale bar, 40 μm. Data represent mean ± SEM. For **b**, **e**, **g,** and **k**, statistical significance was analyzed using two-tailed unpaired Student’s *t*-test. For **c**, statistical significance was analyzed using Pearson correlation analysis.

TALDO1, a key rate-limiting enzyme in the PPP, could be detected in the adult mouse brain, including the cortex and hippocampus, regions that are closely associated with AD pathology (Fig. S2a). A comparison of cultured primary neurons, microglia, and astrocytes showed that TALDO1 was expressed predominantly in neurons (Fig. S2d). Immunofluorescence confirmed its localization in the cytosol, nucleus, axons, and dendrites of primary neurons (Fig. S2e). Further, Western blotting revealed that the TALDO1 protein level was markedly downregulated in both the crude synaptosomal fractions and the homogenates of the temporal cortex of AD patients (Fig. 2a, b), validating the results of proteomic analysis. Furthermore, the TALDO1 protein level negatively correlated with Braak stage (Fig. 2c). This downregulation of TALDO1 was also consistently observed in AD mice. In 2.5-month-old 5 × FAD mice, which exhibit early pathological changes without significant Aβ plaques or cognitive deficits, TALDO1 expression was significantly reduced in the hippocampus and cortex by immunofluorescence staining compared to age-matched WT mice (Fig. S2a–c). Further analysis of cortical synaptosomal fractions also demonstrated significant reductions (Fig. 2d, e). Further, this reduction persisted with age (Fig. 2d, e, Fig. S2f, g).

Similar results were observed in AD model cells. After treating primary neurons with Aβ_42_ oligomers at concentrations of 200 nM and 500 nM for 48 h, the expression of TALDO1 decreased in a dose-dependent manner (Fig. S2h, i). Additionally, in N2A-APPswe cells, which overexpress the Swedish mutation of amyloid precursor protein (APPswe), TALDO1 expression was significantly downregulated at both mRNA and protein levels compared to N2A cells (Fig. S2j–l). These data suggest that the TALDO1 decrease may be associated with soluble Aβ in the early stages of AD. Moreover, TALDO1 was co-localized with the neuronal marker NeuN (Fig. 2f, h). Further analysis demonstrated that TALDO1 was specifically downregulated in neurons within the CA1, CA3, and dentate gyrus (DG) regions of hippocampus and cortex of 5 × FAD mice compared to the WT mice (Fig. 2f, g), with no significant changes observed in astrocytes or microglia (Fig. 2i–k).

Other key enzymes in the PPP, G6PD and TKT, were also detected in neurons of 2.5-month-old 5 × FAD mice, but their expression level did not show significant differences from the WT mice (Fig. S3a–d). These findings manifest that TALDO1 as the primary enzyme of the PPP, was downregulated in neurons of 5 × FAD mice.

### Knockdown of *Taldo1* suppresses glucose metabolism and affects metabolic homeostasis in neurons

**Fig. 3:**
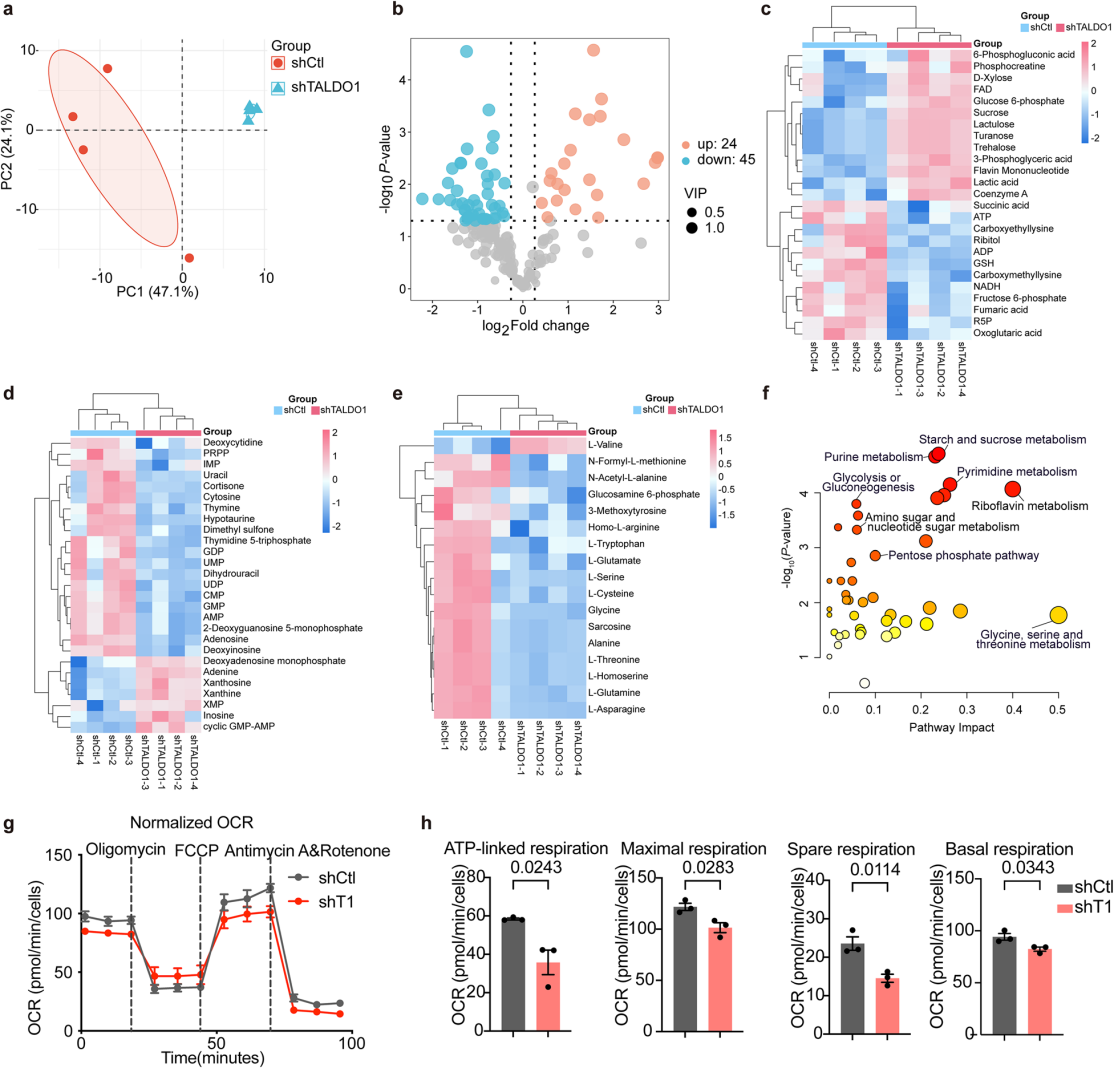
Knockdown of *Taldo1* suppresses the glucose metabolism and impairs metabolic homeostasis. **a** PCA of metabolomic profiles showing clear separation between control and TALDO1–knockdown primary neurons (*n* = 4). **b** Volcano plot displaying significantly altered metabolites. Fold Change > 1.2 or < 0.8, *P*-value < 0.05. **c–e** Heatmaps showing altered metabolites in: Glycolysis and energy metabolism (**c**), Nucleotide biosynthesis and salvage (**d**), and Amino acid metabolism (**e**). **f** KEGG pathway enrichment analysis demonstrating significant enrichment in glycolysis/gluconeogenesis, pentose phosphate pathway, amino sugar and nucleotide sugar metabolism, purine metabolism, and pyrimidine metabolism. **g** Oxygen consumption rates (OCR) measured in mitochondria of primary neurons (*n* = 3 independent replicates). **h** Quantification of ATP-linked respiration, maximal respiration, spare respiration and basal respiration based on OCR (*n *= 3 independent replicates). Data represent mean ± SEM. For **h**, statistical significance was analyzed using two-tailed unpaired Student’s *t*-test

We next investigated whether down-regulation of TALDO1 affects the PPP and even other metabolic pathways, by pseudotargeted metabolomic profiling in primary neurons. The efficiency of *Taldo1* knockdown with *Taldo1* shRNA (ShT1) was confirmed through Western blotting (Fig. S4a, b). PCA analysis revealed a clear separation between neurons with and without *Taldo1* knockdown, indicating a distinct metabolic signature associated with *Taldo1* knockdown (Fig. 3a). Volcano plot analysis identified 69 significantly altered metabolites, including 24 upregulated and 45 downregulated species (Fig. 3b, Table S3 for original metabolic data). Heatmap clustering further demonstrated coordinated metabolic remodeling, particularly in pathways associated with glucose utilization, amino acid metabolism, and nucleotide turnover (Fig. 3c–e). Specifically, R5P and GSH, which are produced or maintained through the PPP, were reduced, along with the downregulation of the downstream metabolite ribitol, suggesting impairment of the PPP flux (Fig. 3c). In parallel, TCA cycle intermediates including α-ketoglutarate (oxoglutaric acid), succinate, and fumarate were markedly decreased. Concomitantly, lactate levels were elevated. These data indicate compromised mitochondrial oxidative metabolism. R5P conversion to phosphoribosyl diphosphate is the initial step for de novo nucleotide synthesis. The markedly decreased phosphoribosyl diphosphate as well as pyrimidine and purine nucleotides (including CMP, UMP, UDP, AMP, IMP and GMP), and accumulation of nucleotide bases and salvage intermediates (such as adenine, inosine, xanthine, and xanthosine), demonstrated that the PPP impairment led to disrupted nucleotide metabolism (Fig. 3d). In addition, key amino acids essential for neuronal antioxidant defense and biosynthesis—including cysteine (rate-limiting for GSH synthesis), serine and glycine (one-carbon donors for antioxidant defense and nucleotide synthesis), and glutamine/glutamate (central to redox buffering and neurotransmission), were significantly reduced (Fig. 3e). Pathway enrichment analysis highlighted significant enrichment in pyrimidine metabolism, purine metabolism, PPP, and glycolysis/gluconeogenesis, as well as amino sugar and nucleotide sugar metabolism, further confirming the disruption of broad metabolic homeostasis by *Taldo1* knockdown (Fig. 3f).

As the TCA cycle was affected, we next investigated whether oxidative phosphorylation was affected through OCR. OCR measurement demonstrated that basal respiration, maximal respiration, ATP-linked respiration, and spare respiratory capacity were all markedly reduced following *Taldo1* knockdown (Fig. 3g, h). HT22 cells with *Taldo1* knockdown by two independent shRNAs (Fig. S4c, d) yielded consistent results (Fig. S4e, f).

Taken together, these findings reveal that neuronal TALDO1 loss reduced glucose metabolism by inhibiting PPP, TCA cycle, and oxidative phosphorylation, thus reducing energy supply and causing broad metabolic collapse characterized by impaired nucleotide synthesis and amino acid depletion. These data highlight TALDO1 as an essential regulator of neuronal carbon metabolism and metabolic homeostasis.

### Knockdown of *Taldo1 *weakens oxidative defense and impairs mitochondrial function

**Fig. 4:**
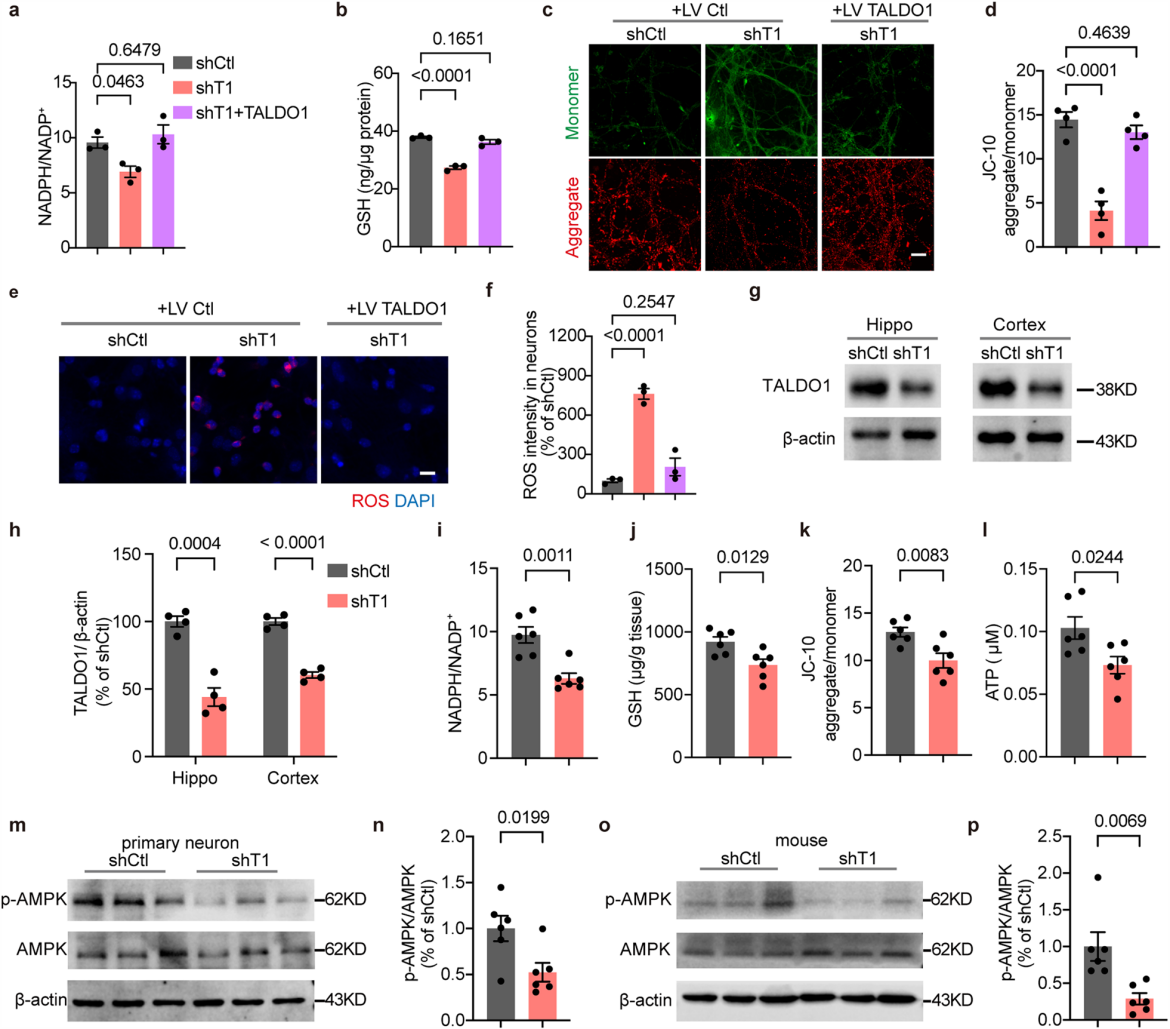
Knockdown of *Taldo1* weakens oxidative defense, impairs mitochondrial function and damages energy sensing **a, b** NADPH/NADP^+^ ratio (**a**) and GSH content (**b**) in primary neurons (*n* = 3 independent replicates). **c, d** Mitochondrial membrane potential measured by JC-10 staining (**c**) and quantification of JC-10 aggregate/monomer ratio (**d**) in primary neurons (*n* = 4 slices with 6 views in each slice analyzed). Scale bar, 40 μm. **e, f** Confocal images (**e**) and quantification (**f**) of ROS content in primary neurons (*n* = 3 slices with 6 views in each slice analyzed). Scale bar, 40 μm. **g, h** Western blot analysis (**g**) and quantification (**h**) of TALDO1 expression in the hippocampus and cortex of mice 8 weeks after virus injection (*n* = 4). **i, j** NADPH/NADP^+^ ratio (**i**) and GSH content (**j**) in the cortex of mice injected with AAVs expressing shT1 or shCtl (*n* = 6). **k** Mitochondrial membrane potential measured by JC-10 staining in the mitochondria purified from the cortex of mice (*n* = 6). **l **ATP production in the cortex of mice (*n* = 6). **m, n** Western blot analysis (**m**) and quantification (**n**) of phosphorylated AMPK (p-AMPK) in the primary neurons (*n* = 6). **o, p** Western blot analysis (**o**) and quantification (**p**) of p-AMPK in the hippocampus of mice injected with AAVs expressing shT1 or shCtl (*n* = 6). Data represent mean ± SEM. For **a, b, d,** and **f**, statistical significance was analyzed using one-way ANOVA followed by Dunnett’s post hoc test. For **h–l**, **n**, and **p**, statistical significance was analyzed using two-tailed unpaired Student’s *t*-test

The PPP provides NADPH to maintain the level of GSH, which is critical in neurons, as they are highly susceptible to oxidative stress due to high oxygen consumption. The above metabolomics indicated reduction of GSH by *Taldo1* knockdown. Thus, we measured the levels of NADPH and GSH in conditions of *Taldo1* knockdown and subsequent overexpression in primary neurons (Fig. S5a). The efficiency of both *Taldo1* knockdown and overexpression was confirmed through Western blotting (Fig. S5b, c). NADPH was significantly reduced in the *Taldo1 *knockdown condition, as manifested by decreased NADPH/NADP^+^ ratio (Fig. 4a). As expected, GSH level was also reduced in neurons with *Taldo1 * knockdown (Fig. 4b). Restoration of TALDO1 expression effectively rescued NADPH and GSH levels (Fig. 4a, b). These results indicated that TALDO1 is essential for maintaining redox homeostasis and thereby strengthening the antioxidant defense. As primary generators of ROS, mitochondria are also vulnerable to oxidative attack. Consistent with the above OCR measurement which indicated mitochondrial respiration was affected, *Taldo1* knockdown significantly reduced mitochondrial membrane potential (Fig. 4c, d), indicating the impairment of mitochondrial function. Both depletion of GSH and mitochondrial dysfunction can increase peroxide products, and as expected, intracellular ROS level was significantly elevated (Fig. 4e, f). Restoration of TALDO1 expression effectively rescued mitochondrial membrane potential and ROS accumulation to near control levels (Fig. 4c–f). Consistently, similar results were observed in HT22 cells, as* Taldo1 * knockdown using two independent shRNAs impaired antioxidant capacity, decreased mitochondrial function, and increased ROS accumulation (Fig. S5d–i).

To further elucidate the role of TALDO1, we microinjected adenoviruses expressing shT1 under synapsin-1 (Syn1) promoter into the bilateral ventricles of WT mice to knock down neuronal *Taldo1* (Fig. S6a and Fig. 4g, h). Consistent with the in vitro results, decreases of NADPH and GSH levels as well as mitochondrial membrane potential were detected in the cerebral cortex of mice with *Taldo1* knockdown (Fig. 4i–k). Moreover, knockdown of neuronal *Taldo1* significantly reduced ATP production (Fig. 4l), confirming that *Taldo1 *preserves redox homeostasis and energy supply.

AMP-activated protein kinase (AMPK) functions as a critical sensor of intracellular energy status to maintain metabolic balance and mitochondrial function [50, 51]. To further explore the effect of TALDO1 on neuronal energy sensing, we examined AMPK activity by measuring the level of phosphorylated AMPK. A marked reduction in phosphorylated AMPK was observed in both primary neurons and hippocampus of mice with *Taldo1* knockdown (Fig. 4m–p).

Collectively, downregulation of TALDO1 results in NADPH depletion, which compromises the redox balance and mitochondrial function, and decreased AMPK phosphorylation, which further weakens the energy-sensing capacity and adaptive metabolic responses of neurons.

### Neuronal *Taldo1* knockdown impairs neuronal structure and synaptic integrity

**Fig. 5:**
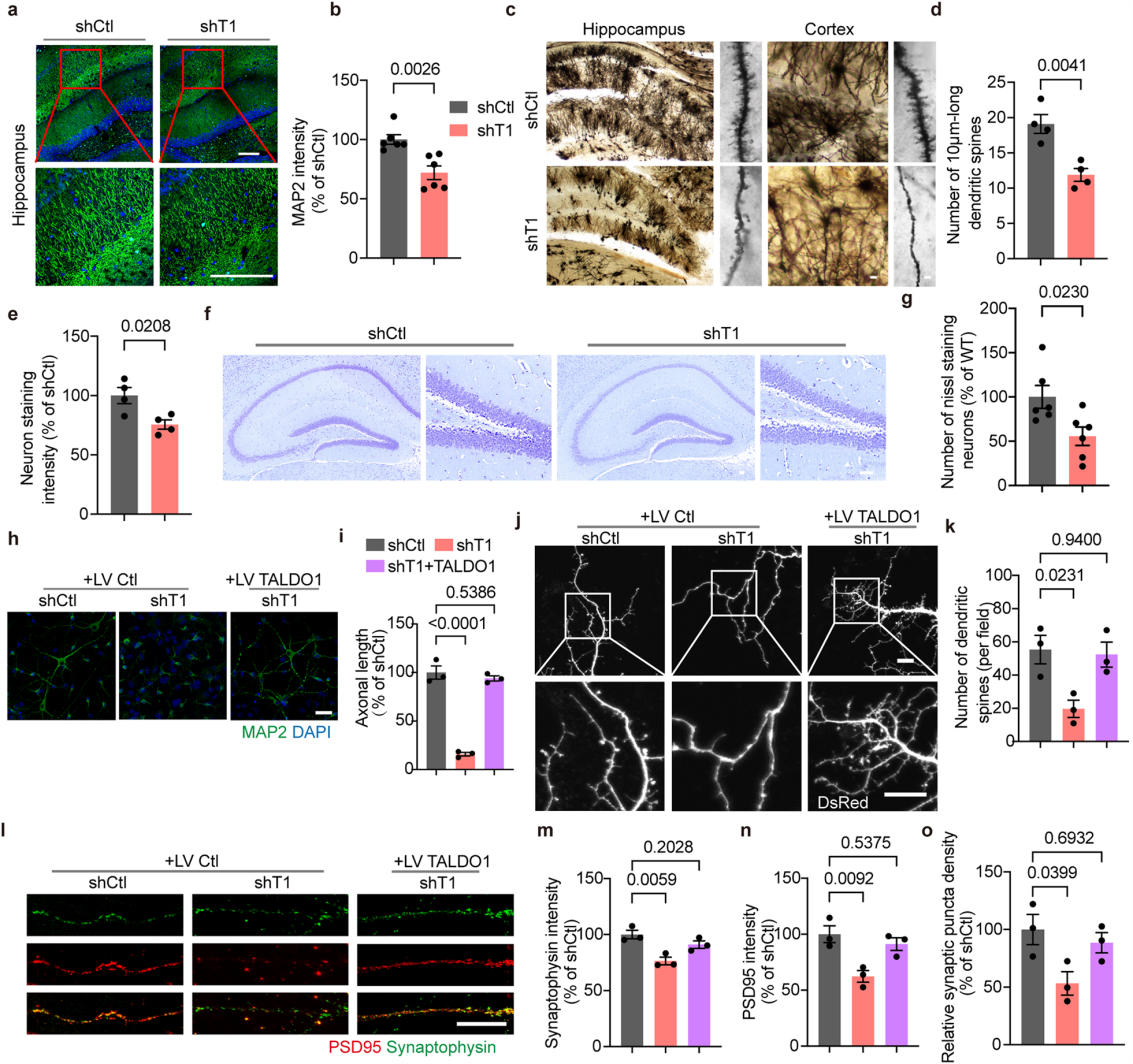
Knockdown of *Taldo1* impairs neuronal morphology and synaptic integrity **a**, **b** Confocal images (**a**) and quantification (**b**) of the structural integrity of the neuronal cytoskeletal protein MAP2 in the hippocampus of mice (*n* = 6). The boxed areas were zoomed in the under panels. Scale bar, 200 μm. **c**-**e** Representative images (**c**) and quantification of dendritic spine number (**d**) and neuronal density (**e**) of Golgi-stained dendrites in the hippocampus and cortex of mice (*n* = 4). Scale bar, 200 μm (left panel), 1 μm (right panel). **f**, **g** Representative images (**f**) and quantification (**g**) of Nissl staining in the hippocampus (*n* = 6 slices from 3 mice per group). Scale bar, 10 μm. **h**, **i** Confocal images (**h**) and quantification (**i**) of the structural integrity of the neuronal cytoskeletal protein MAP2 in primary neurons (*n* = 3). Scale bar, 40 μm. **j**, **k** Confocal images of neuronal dendritic spines (**j**) and quantification (**k**) of dendritic spine density in primary neurons (*n* = 3 slices with 6 views in each slice analyzed). The boxed areas were zoomed in the under panels. Scale bars, 40 μm. **l**–**o** Confocal images of pre- and post-synaptic markers in primary neurons (**l**), and quantification of synaptophysin (**m**) and PSD95 (**n**), and quantification of relative synaptic puncta density defined by the colocalization of synaptophysin and PSD95 (**o**). (*n* = 3 slices with 6 views in each slice analyzed). Scale bar, 10 μm. Data represent mean ± SEM. For **b**, **d**, **e**, and **g**, statistical significance was analyzed using two-tailed unpaired Student’s *t*-test. For **i**, **k**, and **m–o**, statistical significance was analyzed using one-way ANOVA followed by Dunnett’s post hoc test

To investigate whether the metabolic changes induced by *Taldo1* knockdown would ultimately affect neuronal structure and synaptic integrity, we analyzed dendritic architecture and synaptic protein levels in both mouse brain tissues and primary neurons. We found that *Taldo1* knockdown reduced MAP2 immunoreactivity in the hippocampus, indicating impaired dendritic branching and cytoskeletal integrity (Fig. 5a, b). Golgi staining revealed a decrease in dendritic spine density, reflecting compromised synaptic connectivity (Fig. 5c–e). Nissl staining further showed a decrease in both the number and the staining intensity of Nissl bodies in the hippocampus of mice with *Taldo1* knockdown, indicative of neuronal loss and morphological abnormalities (Fig. 5f, g).

Parallel analyses in primary neurons confirmed that *Taldo1* knockdown markedly decreased dendritic arborization and spine density, as visualized by MAP2 immunofluorescence and DsRed labeling (Fig. 5h–k). Furthermore, the expression of synaptic proteins, including the presynaptic marker synaptophysin and the postsynaptic marker PSD95, was significantly reduced in TALDO1-deficient neurons (Fig. 5l–n). Consistently, the density of synaptic puncta, defined as the colocalization of synaptophysin and PSD-95, was markedly decreased in TALDO1-deficient neurons (Fig. 5o). Restoration of TALDO1 expression rescued dendritic complexity, spine density, synaptic protein levels, and active synapses to near-control levels (Fig. 5h–o). These data demonstrate that TALDO1 is essential for maintaining neuronal morphology and synaptic integrity.

### TALDO1 is crucial for cognitive function and synaptic plasticity

**Fig. 6:**
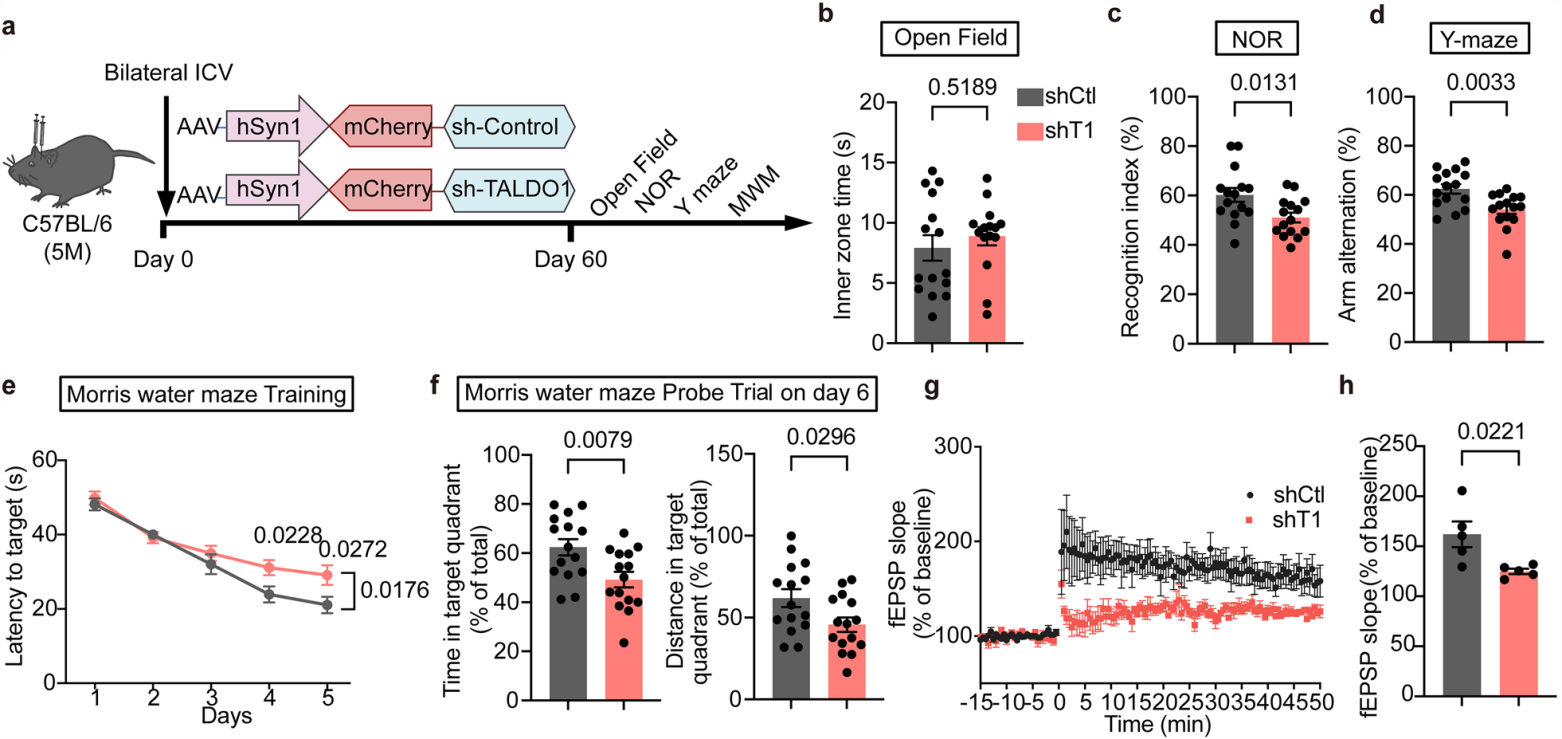
Knockdown of *Taldo1* impairs cognitive function and disrupts synaptic plasticity in WT mice. **a** Schematic showing stereotaxic injection of AAVs expressing shT1 or the shCtl with mCherry in 5-month-old WT mice. Cognitive function was assessed 2 months post-injection. **b**–**f** Open Field (**b**), New Objects Recognition (**c**), Y maze (**d**) and MWM tests (**e**, **f**) were performed to assess cognitive function (*n* = 15). **g**, **h** Recordings of fEPSP in the hippocampus after θ burst stimulation (**g**) and average fEPSP slope during the last 10 min (**h**) in mice (*n* = 5). Data represent mean ± SEM. For **c**, **d**, **f**, and** h**, statistical significance was analyzed using two-tailed unpaired Student’s *t*-test. For **b**, statistical significance was analyzed using the Mann–Whitney test due to non-normal distribution. For **e**, statistical significance was analyzed using two-way ANOVA followed by Fisher’s LSD post hoc test

To determine whether the reduction of TALDO1 in neurons influences behavior, we assessed the memory and spatial learning abilities of 5-month-old WT mice with neuronal knockdown of *Taldo1* for 2 months (Fig. 6a and Fig. S6a). Notably, TALDO1 downregulation had no significant effect on body weight (Fig. S6b). The open field test demonstrated that TALDO1 downregulation did not alter locomotor activity (Fig. S6c, d) or anxiety-like behavior (Fig. 6b). To evaluate cognitive function, recognition memory was first examined using the NOR test, followed by the Y-maze and MWM tests to assess hippocampus-dependent spatial learning and memory. In the NOR test, *Taldo1* knockdown resulted in a decreased discrimination index 24 h after training (Fig. 6c), indicating impairment in recognition memory. In the Y-maze, *Taldo1* knockdown attenuated the spontaneous alteration behavior of mice without affecting the total entries (Fig. 6d and Fig. S6e). In the MWM tests, mice with *Taldo1* knockdown exhibited impaired learning, as evidenced by increased latency to target the platform during the 5-day training phase, compared to the mice injected with control viruses (Fig. 6e). Furthermore, during the probe trial, mice with *Taldo1* knockdown spent significantly less time and traveled a shorter distance in the target quadrant (Fig. 6f and Fig. S6g). The average swimming speed during the test did not differ between the groups (Fig. S6f), indicating that *Taldo1* knockdown had no effect on locomotor function.

To evaluate synaptic plasticity, we induced LTP in hippocampal slices. LTP was quantified by measuring the percentage change in the fEPSP slope over time. Mice with *Taldo1* knockdown exhibited significantly reduced LTP compared to control mice, as evidenced by decreases in the fEPSP slope (Fig. 6g, h). These findings indicate that TALDO1 is essential for maintaining synaptic plasticity and cognitive function.

### Restoration of TALDO1 expression improves glucose metabolism, and restores redox balance and mitochondrial function in AD mice

The above data indicate that TALDO1 plays an essential role in glucose metabolism and maintains neuronal redox balance and mitochondrial function, thus affecting the neuronal integrity and cognition. Given that impaired glucose metabolism, oxidative stress, and mitochondrial dysfunction are key pathological features of AD [17, 21, 52–56], we next examined whether neuronal TALDO1 downregulation contributes to these metabolic defects in AD brains.

**Fig. 7:**
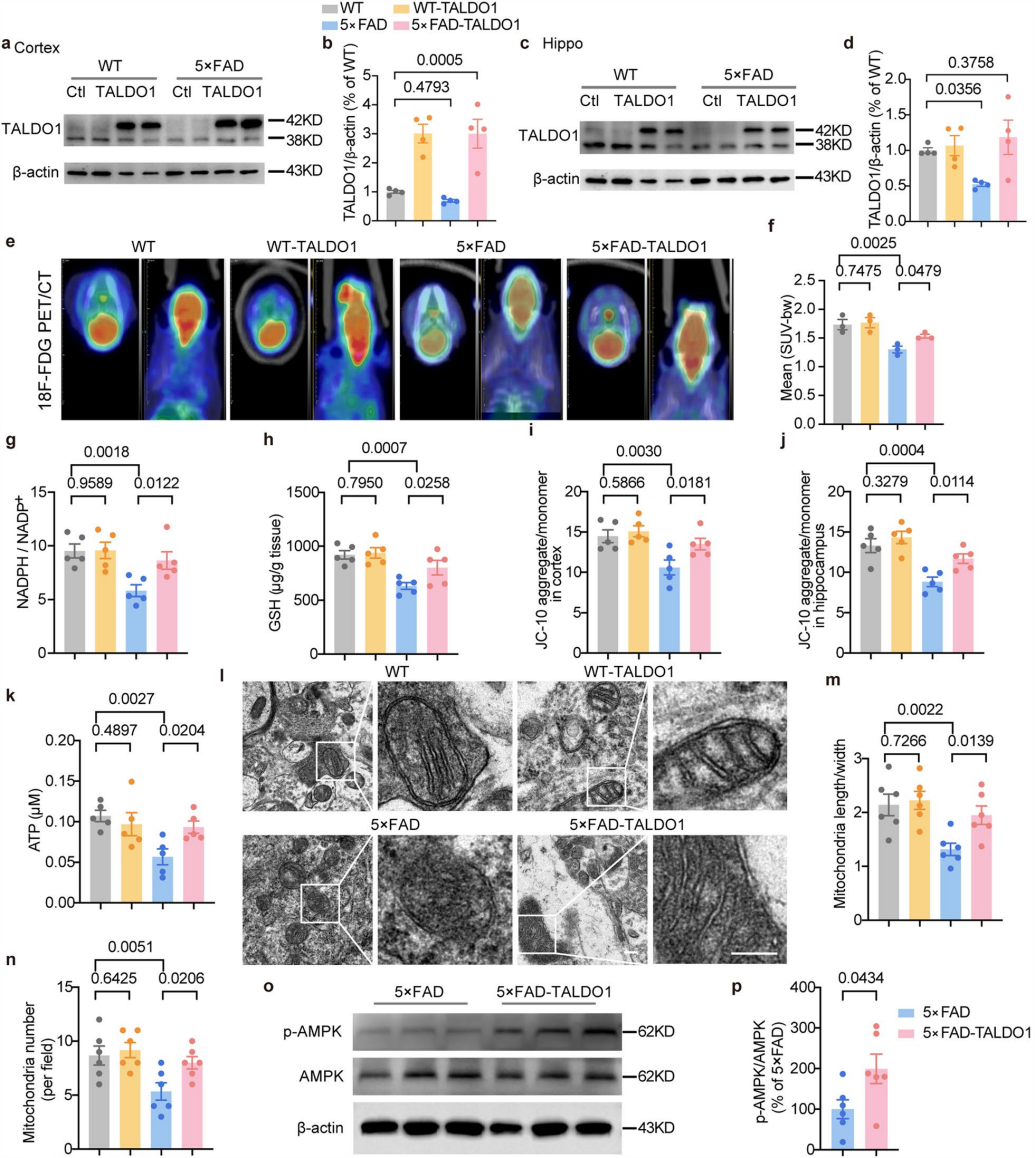
Restoration of neuronal TALDO1 expression alleviates PPP-mediated redox imbalance and restores metabolic homeostasis in 5 × FAD mice **a**-**d** Western blot of TALDO1 in the cortex (**a, b**) and hippocampus (**c, d**) of WT and 5 × FAD mice 45 days after lentiviral injection (*n* = 4) . **e, f** ^18^F-FDG PET/CT detection of glucose uptake (**e**) and analysis of standard uptake values for glucose (**f**) in the cortex (*n* = 3). **g, h** NADPH/NADP⁺ ratio (**g**) and GSH content (**h**) in the cerebral cortex of WT and 5 × FAD mice after lentiviral injection to restore the expression of TALDO1 in neurons (*n* = 5). **i, j** Mitochondrial membrane potential measured by JC-10 aggregate/monomer fluorescence ratio in isolated mitochondria from the cortex (**i**) and hippocampus (**j**) of mice (*n* = 5). **k** ATP production in the cortex of mice (*n* = 5). **l**-**n** Mitochondrial electron micrographs in the hippocampus of mice (**l**), with quantification of mitochondrial length/width (**m**) and number (**n**) (*n* = 6 slices from 3 mice per group). The boxed areas are zoomed in the right panels. Scale bar, 0.5 μm. **o, p** Western blot analysis (**o**) and quantification (**p**) of phosphorylated AMPK (p-AMPK) in the cortex of 5 × FAD mice (*n* = 6). Data represent mean ± SEM. For **b, d, f-k, m**, and **n**, statistical significance was analyzed using two-way ANOVA followed by Fisher’s LSD post hoc test. For **p**, statistical significance was analyzed using two-tailed unpaired Student’s *t*-test

To address this question, we restored neuronal TALDO1 expression in 5 × FAD mice. Injection of lentiviruses expressing *Taldo1* under the Syn1 promotor into the bilateral ventricles of 5 × FAD mice resulted in significant upregulation of TALDO1 expression in the hippocampus and cortex of 5 × FAD mice (Fig. 7a–d, and Fig. S7a). PET/CT imaging with ^18^F-FDG showed that the 5 × FAD mice exhibited significantly lower cortical glucose uptake compared to the WT mice (Fig. 7e, f). Conversely, neuronal overexpression of TALDO1 in 5 × FAD mice restored the cortical glucose uptake (Fig. 7e, f). Moreover, the levels of NADPH and GSH were elevated in 5 × FAD mice with restoration of neuronal TALDO1 expression (Fig. 7g, h), indicating strengthening of the oxidative defense. Furthermore, mitochondrial dysfunction in AD was also reversed, reflected by improved mitochondrial membrane potential and energy production in the cortex or hippocampus of 5 × FAD mice with restoration of neuronal TALDO1 expression (Fig. 7i–k). The number and the morphology of mitochondria were also ameliorated. The mitochondria in the hippocampus of 5 × FAD mice showed a decreased number and a more oval shape with indistinct cristae compared to those in WT mice (Fig. 7l–n). In contrast, restoration of neuronal TALDO1 expression increased the number of mitochondria, with elongated or elliptical shapes and clearer cristae (Fig. 7l–n). These results demonstrate that neuronal TALDO1 replenishment restores the redox homeostasis and mitochondrial integrity in AD. Restoration of neuronal TALDO1 expression also enhanced AMPK activity in 5 × FAD mice, as evidenced by increased phosphorylation of AMPK (Fig. 7o, p), indicating the recovery of energy sensing by AMPK.

### Restoration of neuronal TALDO1 expression rescues neuronal damage and cognitive dysfunction in AD mice

**Fig. 8:**
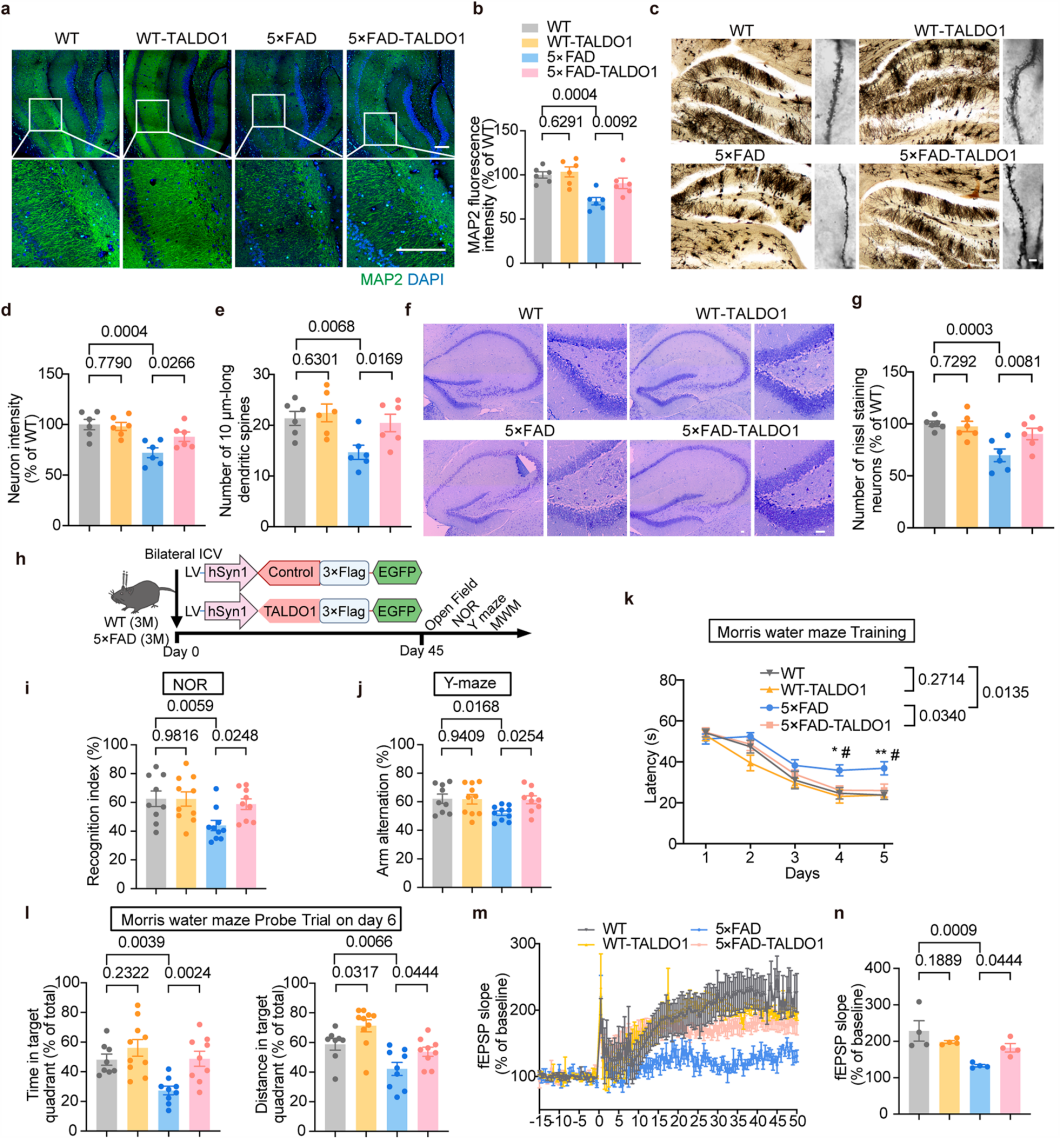
Restoration of TALDO1 expression improves neuronal morphology, synaptic integrity and cognitive function in 5 × FAD mice **a**, **b** Confocal images (**a**) and quantification (**b**) of MAP2 (green) in hippocampus of WT and 5 × FAD mice 45 days after lentiviral injection (*n *= 6 slices from 3 mice per group). The boxed areas are zoomed in the under panels. Nuclei were stained with DAPI (blue). Scale bar, 200 μm. **c**–**e** Representative images (**c**) and quantification of neuron density (**d**) and dendritic spine number (**e**) of Golgi-stained dendrites in the hippocampus (*n* = 6 slices from 3 mice per group). Scale bars, 100 μm (left panels), 1 μm (right panels). **f**, **g** Representative images (**f**) and quantification (**g**) of Nissl staining in the hippocampus (*n* = 6 slices from 3 mice per group). Scale bars, 10 μm. **h** Schematic of the experimental strategy: 3-month-old WT and 5 × FAD mice were injected with lentiviruses expressing TALDO1 or the corresponding controls. Cognitive function was tested 45 days later. **i**–**l** Behavioral assessment of by NOR (**i**), Y maze (**j**) and MWM tests (**k**, **l**) (*n* = 9 for WT and 5 × FAD-TALDO1, *n* = 10 for WT-TALDO1 and 5 × FAD in NOR and Y maze; *n* = 8 for WT, *n* = 10 for WT-TALDO1, *n* = 9 for 5 × FAD and 5 × FAD-TALDO1 in MWM). *P = 0.0110 between WT and 5 × FAD, and ^#^*P* = 0.0114 between 5 × FAD and 5 × FAD-TALDO1 on Day 4. **P = 0.0048 between WT and 5 × FAD, and ^#^*P* = 0.0293 between 5 × FAD and 5 × FAD-TALDO1 on Day 5 in **k**. **m**, **n** Recordings of fEPSP in the hippocampus after θ burst stimulation (**m**) and average fEPSP slope during the last 10 min (**n**) (*n* = 4). Data represent mean ± SEM. For **b**, **d**, **e**, **g**, **i**, **j**, **l** and **n**, statistical significance was analyzed using two-way ANOVA followed by Fisher’s LSD post hoc test. For **k**, statistical significance was analyzed using two-way ANOVA followed by the original FDR correction (Benjamini–Hochberg) to compare differences between groups, and three-way ANOVA followed by Fisher’s LSD post hoc test to assess differences at various time points

Next, we assessed the impact of neuronal TALDO1 restoration on neuronal integrity in AD mice. Golgi staining and MAP2 immunofluorescence demonstrated a reduction in the number of hippocampal neurons and dendritic spine density in 5 × FAD mice; whereas restoration of TALDO1 expression improved both neuronal morphology and dendritic spine density (Fig. 8a–e). Nissl staining showed neuronal loss and morphological abnormalities in the hippocampus of 5 × FAD mice, while restoration of TALDO1 expression increased both the quantity and the staining intensity of Nissl bodies, indicating the recovery of neurons (Fig. 8f, g).

We next examined whether neuronal TALDO1 restoration could ameliorate cognitive deficits in AD mice (Fig. 8h). In the NOR test (Fig. 8i), Y-maze test (Fig. 8j), and MWM test (Fig. 8k, l, and Fig. S7h), restoration of TALDO1 expression in neurons significantly rescued cognitive impairments in 5 × FAD mice, without affecting the body weight (Fig. S7b), locomotor activity (Fig. S7c, d, f, g), or anxiety-like behavior (Fig. S7e). Moreover, electrophysiological recordings showed a significant improvement in LTP deficits in 5 × FAD mice with restored TALDO1 expression in neurons (Fig. 8m, n).

Taken together, these findings strongly indicate that restoration of neuronal TALDO1 expression effectively rescues neuronal integrity and ameliorates cognitive dysfunction in AD mice.

### Neuronal expression of TALDO1 reduces Aβ deposition

**Fig. 9:**
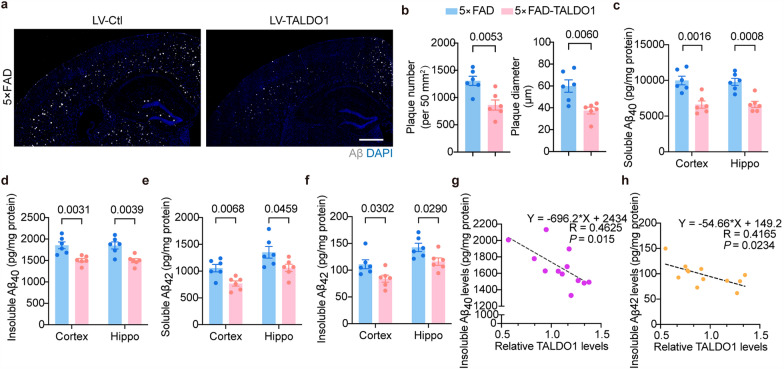
Neuronal TALDO1 reduces Aβ deposition in 5 × FAD mice. **a**, **b** Confocal images of Aβ plaque (**a**) and quantification of the number and size of Aβ plaques (**b**) in the hippocampus and cortex of 5 × FAD mice after restoration of neuronal expression of TALDO1 (*n* = 6 slices from 3 mice per group). Scale bar, 500 μm. Nuclei were stained with DAPI (blue). **c**-**f** Soluble and insoluble Aβ_40_ (**c**, **d**) and Aβ_42_ levels (**e**, **f**) in cortex and hippocampus of 5 × FAD mice (*n* = 6). **g**, **h** The correlation between TALDO1 expression level and insoluble Aβ_40_ (**g**) or Aβ_42_ (**h**) levels in 5 × FAD mice was assessed using linear regression (*n* = 6). Data represent mean ± SEM. For **b**-**f**, statistical significance was analyzed using two-tailed unpaired Student’s *t*-test. For **g**, **h**, statistical significance was analyzed using Pearson correlation analysis

We further investigated whether neuronal TALDO1 could affect Aβ deposition. Neuronal expression of TALDO1 in 5 × FAD mice significantly reduced both the number and the size of Aβ plaques in the cortex and hippocampus (Fig. 9a, b). Further analysis confirmed that both soluble and insoluble levels of Aβ_40_ and Aβ_42_ were significantly reduced in the brains of TALDO1-expressing 5 × FAD mice (Fig. 9c–f). Consistently, a negative correlation was observed between TALDO1 expression and levels of insoluble Aβ_40_ and Aβ_42_ (Fig. 9g, h) in the 5 × FAD mice. These results further demonstrate that restoration of neuronal expression of TALDO1 exerts a protective role in AD pathology.

## Discussion

AD is characterized by progressive neurodegeneration associated with metabolic dysfunction and mitochondrial impairment [54, 57, 58]. Neurons rely heavily on optimal glucose metabolism and mitochondrial function to maintain redox balance and support synaptic activity [9, 55, 56]. In the current study, our synaptic proteomic analysis revealed that proteins involved in glucose metabolism were the most profoundly dysregulated in AD, underscoring the centrality of glucose metabolism in synaptic function and its critical involvement in AD pathogenesis. Notably, among these altered proteins, we identified TALDO1, a key enzyme of the non-oxidative branch of the PPP, to be significantly downregulated early in disease progression. Reduced expression of neuronal TALDO1 precipitated the inhibition of PPP, TCA cycle, and oxidative phosphorylation, driving broad metabolic collapse. TALDO1 is indispensable for maintaining NADPH and GSH pools that protect mitochondria from oxidative damage, thereby protecting synaptic integrity. Restoration of TALDO1 expression in AD models normalized glucose uptake, rebalanced redox status, and revitalized energy supply, which in turn rescued synaptic plasticity, cognitive deficits and Aβ pathology. Therefore, our study provided compelling evidence that TALDO1 is instrumental in modulating neuronal glucose metabolism and metabolic homeostasis.

Glycolysis and the PPP represent divergent, competing cytosolic pathways that process glucose for different purposes. Reducing equivalents derived from glucose and other fuels are transferred to the mitochondrial electron transport chain, where they drive oxidative phosphorylation and ATP synthesis. In neurons, weak glycolysis caused by low activity of PFK1, which results in preference for the PPP, is required for maintaining functions of neurons [17, 59]. The PPP not only generates NADPH and GSH to maintain redox balance and provides critical precursors for biosynthetic pathways, but also supplies pyruvate to fuel oxidative phosphorylation for ATP production [15]. Moreover, its non-oxidative branch through TKT and TALDO1 provides crucial metabolic flexibility by interconverting glucose back into glycolytic intermediates. Our findings provide direct experimental evidence supporting this model. We demonstrated that downregulation of TALDO1, a key PPP enzyme, not only impairs the PPP itself but also precipitates a concurrent suppression of TCA cycle and oxidative phosphorylation, which further weakens oxidative defense and lowers energy supply, thus damaging neuronal functions. These data highlight the central role of the PPP in neuronal viability, indicating that the impairment of the PPP constitutes a critical mechanism in the pathogenesis of AD.

It has long been established that the flux through the PPP is downregulated in AD; however, the specific enzymatic determinants underlying this impairment remain incompletely characterized. Previous studies have documented both downregulation and upregulation of G6PD, the rate-limiting enzyme that diverts glucose into the PPP in AD. Similarly, the role of other enzymes, such as TKT which is linked with thiamine deficiency, remains incompletely understood. However, there is a growing body of literature on the alterations of TALDO1 in AD. A study with bioinformatic analysis suggested that TALDO1 may serve as a potential hub gene in AD [60]. Another proteomic analysis revealed downregulation of TALDO1 in the brains of AD patients [58]. A recent study with RNA-Seq analysis on synaptosomal mRNA also revealed down-regulation of TALDO1, without identification of G6PD and TKT [61]. Our data also demonstrated no significant alteration of G6PD and TKT expression in 2.5-month-old 5 × FAD mice. Further, our data demonstrated that TALDO1 was significantly downregulated in the early stages of AD. These findings implicate that TALDO1 may serve as a key pathogenic factor underlying the observed impairment of the PPP.

TALDO1 deficiency is a genetic disease characterized by an early onset of symptoms in childhood. Depletion of NADPH, oxidative damage, and compromised mitochondrial function have been reported [16, 32, 33, 62–64]. However, there is no report of cognitive impairment, with one study showing that some patients exhibit mild intellectual disability or motor slowing in TALDO1 deficiency [65]. Thus, the direct relationship between neuronal TALDO1 and cognitive impairment has not been established. Therefore, for the first time, our study manifests a direct link between neuronal TALDO1 and cognitive impairment in adulthood. Furthermore, these data are consistent with observations in AD mouse models, in which flux through PPP and oxidative phosphorylation is significantly reduced, leading to impaired antioxidant capacity and energy metabolism in the brain [18, 52]. Importantly, our finding that downregulation of TALDO1 in neurons leads to impaired synaptic plasticity and cognition is consistent with previous reports that dysfunction of synaptic mitochondria correlates with impaired synaptic function and cognitive deficits in AD [21, 66, 67].

Here, we also found that restoring neuronal expression of TALDO1 in 5 × FAD mice reduced Aβ deposition. A recent study indicated that enhancing AMPK signaling in neurons can reduce Aβ generation [68]. Thus, the decreased soluble and insoluble Aβ_40_ and Aβ_42_ and amyloid plaques could be due to the increased AMPK activity induced by TALDO1. Besides, the reduced Aβ deposition can also be attributed to the improved neuronal homeostasis by TALDO1, as the impairment of synaptic function in AD allows Aβ to propagate to various brain regions through trans-synaptic transmission [69].

The specific downregulation of TALDO1 in AD-affected neurons but not in astrocytes and microglia can be attributed to various factors. Distinct types of neural cells exhibit unique glycolytic pathways. As mentioned above, neurons preferentially utilize glucose via the PPP. In contrast, astrocytes primarily produce lactate through glycolysis, serving as a metabolic substrate for neurons [70]. Microglia demonstrate considerable metabolic flexibility, and the shifts in glycolipid metabolism are crucial for immune signaling responses [71]. Additionally, the genetic information and cellular functions differ among various neural cell types, leading to the activation of distinct molecular programs in response to diverse stimuli. For example, while hexokinase 2 is present in multiple neural cell types, it is only upregulated in microglia in the brains of AD patients, with no significant changes observed in astrocytes and neurons [71]. This phenomenon may be attributed to the complex regulatory networks inherent to each neural cell type.

## Conclusions

Our study revealed the pivotal role of glucose metabolism in mediating neuronal dysfunction in AD. Downregulation of TALDO1, a critical enzyme in the PPP, could be a key driver of neuronal glucose metabolic dysfunction in AD. Our data demonstrated that downregulation of TALDO1 impairs both the PPP and the oxidative phosphorylation, causing broad metabolic collapse including reduced energy supply and diminished biosynthetic capacity. The impaired PPP depletes the cellular NADPH and GSH, causing impairment of antioxidant defenses in neurons and leading to oxidative damage, which precipitates mitochondrial dysfunction. This creates a vicious cycle of energy failure and cellular damage that culminates in synaptic loss and cognitive impairment. Restoration of TALDO1 expression improves glucose uptake, maintains neuronal metabolic homeostasis, rescues neuronal function and cognition, and decreases Aβ pathology. Consequently, our findings position TALDO1 decrease as a primary pathogenic factor responsible for the impaired PPP observed in AD. The present study provides a new mechanism for AD pathogenesis and offers a potential therapeutic target for AD.

## Supplementary Information

Additional file 1 **Fig S1**. Extraction of synaptosomes for proteomics. **Fig S2**. The expression pattern of TALDO1 in mouse brain, primary neurons and AD model cells. **Fig S3**. TKT and G6PD expression is not changed in the cortical neurons of 5×FAD mice. **Fig S4**. Neuronal *Taldo1*  knockdown reduces glucose metabolism and disrupts metabolic homeostasis. **Fig S5**. Knockdown of *Taldo1* causes oxidative stress and mitochondrial dysfunction.** Fig S6**. Neuronal knockdown of *Taldo1* in WT mice does not affect body weight and locomotor activity. **Fig S7**. Effects of restoring neuronal expression of TALDO1 on body weight, locomotion, anxiety behavior and neuronal skeleton in 5×FAD mice.

Additional file 2 **Table S1**. Information of human subjects used in the study.

Additional file 3 **Table S2**. Identified proteins from proteomic analysis.

Additional file 4 **Table S3**. Data of untargeted metabolomics.

Additional file 5 Unprocessed western blots.

## Data Availability

Proteomic raw data are deposited in the iProX (https://www.iprox.cn) with Project ID IPX0012529000. Other data supporting the findings of this study are included in this article and Additional files or are available from the corresponding author on reasonable request.

## Abbreviations

AD: Alzheimer’s disease
PPP: Pentose phosphate pathway
TALDO1: Transaldolase 1
Aβ: β-Amyloid
PFK1: Phosphofructokinase-1
NADPH: Nicotinamide adenine dinucleotide phosphate
R5P: Ribose-5-phosphate
G6PD: Glucose-6-phosphate dehydrogenase
TKT: Transketolase
GSH: Glutathione
ROS: Reactive oxygen species
NDC: Non-dementia controls
PCA: Principal component analysis
KEGG: Kyoto encyclopedia of genes and genomes
GSEA: Gene set enrichment analysis
TEM: Transmission electron microscopy
OCR: Oxygen consumption rates
PET/CT: Positron emission tomography/computed tomography
^18^F-FDG: ^18^F-fluorodeoxyglucose
NOR: Novel object recognition
MWM: Morris water maze
fEPSPs: Field excitatory postsynaptic potential slope
LTP: Long-term potentiation
ELISA: Enzyme-linked immunosorbent assay
SEM: Standard error of the mean
DG: Dentate gyrus
Syn1: Synapsin-1
AMPK: AMP-activated protein kinase
