# Supplementary material for: Transaldolase 1 contributes to pentose phosphate pathway disruption and synaptic dysfunction in Alzheimer’s disease
Source: Transl Neurodegener. 2026 Jul 29;15:35. doi: 10.1186/s40035-026-00567-z (PMC13418138; doi:10.1186/s40035-026-00567-z)
Supplement: Supplementary file 1 — Additional file 1 Fig S1. Extraction of synaptosomes for proteomics. Fig S2. The expression pattern of TALDO1 in mouse brain, primary neurons and AD model cells. Fig S3. TKT and G6PD expression is not changed in the cortical neurons of 5×FAD mice. Fig S4. Neuronal Taldo1 knockdown reduces glucose metabolism and disrupts metabolic homeostasis. Fig S5. Knockdown of Taldo1 causes oxidative stress and mitochondrial dysfunction. Fig S6. Neuronal knockdown of Taldo1 in WT mice does not affect body weight and locomotor activity. Fig S7. Effects of restoring neuronal expression of TALDO1 on body weight, locomotion, anxiety behavior and neuronal skeleton in 5×FAD mice. [file 40035_2026_567_MOESM1_ESM.docx]

Supporting Information

Transaldolase 1 contributes to pentose phosphate pathway disruption and synaptic dysfunction in Alzheimer's disease

Xiaoyu Hu, Ying Yu, Haorui Luo, Jiabing Li, Xiaofei Zhang, Gang Wang, Jianping Li, Juan Li, Hongzhuan Chen and Yu Qiu


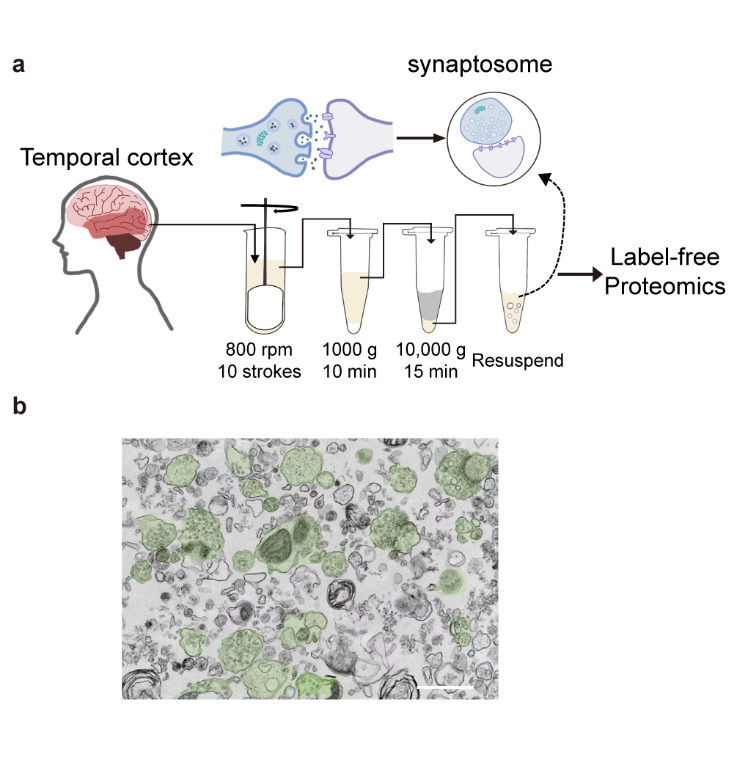


#### Fig. S1 Extraction of synaptosomes for proteomics.

**a** Extraction process for crude synaptosomal compartments. **b** Characterization of isolated synaptosome crude extracts using TEM. The synaptosomes were colored in light green. Scale bar, 1 μm.


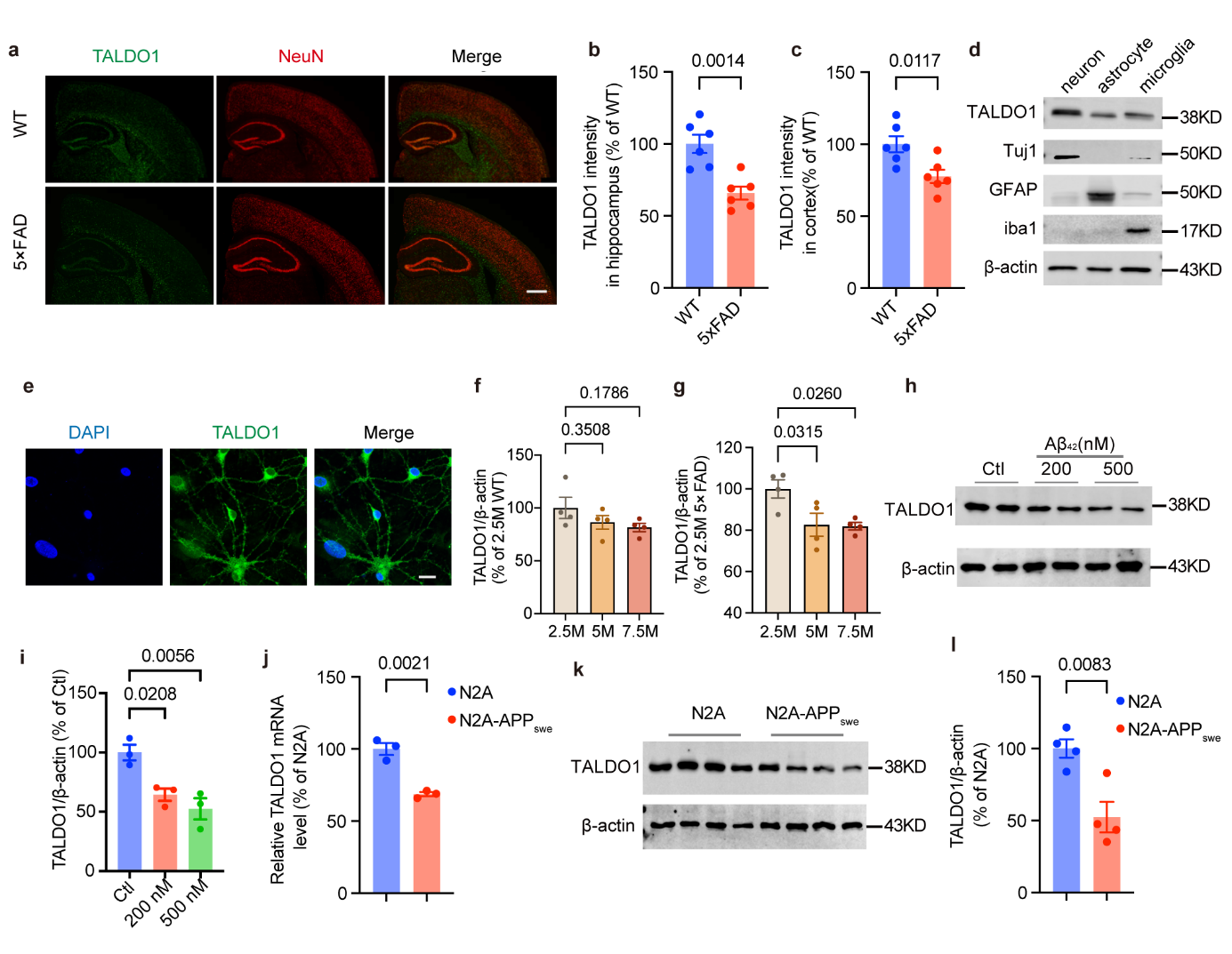


#### Fig. S2 The expression pattern of TALDO1 in mouse brain, primary neurons and AD model cells.

**a**-**c** Confocal images (**a**) and quantification of TALDO1 expression in the cortex (**b**) and hippocampus (**c**) of 2.5-month-old WT and 5×FAD mice (*n* = 6). Scale bar, 500 μm. **d** Expression levels of TALDO1 protein in primary cortical neurons, astrocytes and microglia cultured in vitro. **e** Confocal images showing the distribution of TALDO1 expression in primary cortical neurons from C57BL/6 mouse embryos. Scale bar, 20 μm. **f**, **g** Western blot quantification of TALDO1 in the P2 fraction of brain tissues from WT (**f**) and 5xFAD mice (**g**) at different ages. **h**, **i** Western blot analysis (**h**) and quantification (**i**) of TALDO1 in primary neurons treated with Aβ_42_ oligomers (0 nM, 200 nM, and 500 nM) for 48 h (*n* = 3 indepent replicates). **j** Evaluation of TALDO1 mRNA expression levels in N2A and N2A-APPswe cells (*n* = 3). **k**, **l** Western blot analysis (**k**) and quantification (**l**) of TALDO1 in N2A and N2A-APPswe cells (*n* = 4 indepent replicates). Data represent mean ± SEM. For **b**, **c**, **j** and **l**, statistical significance was analyzed using two-tailed unpaired Student's t-test. For **f**, **g**, and **i** statistical significance was analyzed using one-way ANOVA followed by Dunnett’s post hoc test.


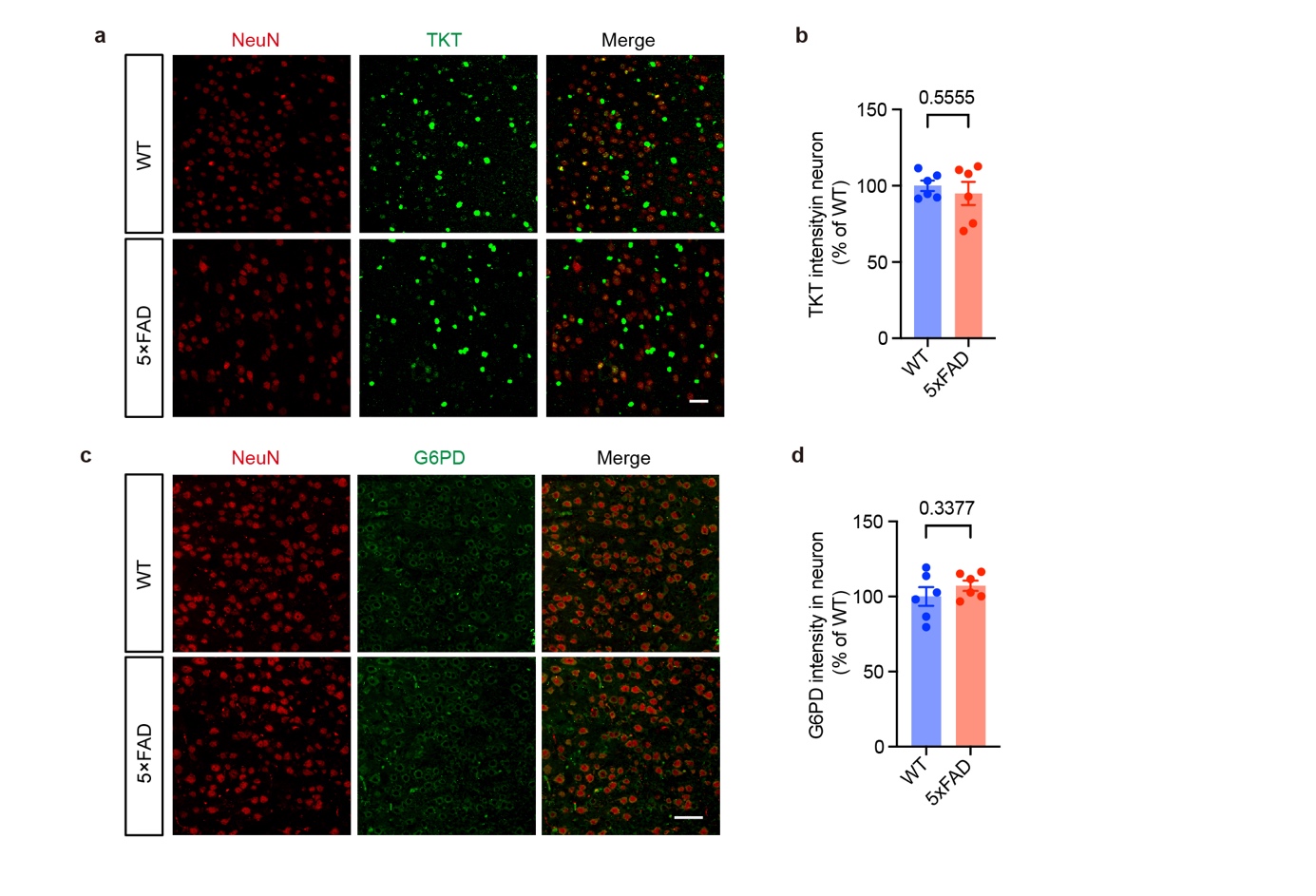


#### Fig. S3 TKT and G6PD expression is not changed in the cortical neurons of 5×FAD mice.

**a**, **b** Confocal images (**a**) and quantification (**b**) of the co-expression of TKT and neuronal marker NeuN in the cortex of 2.5-month-old WT and 5×FAD mice (*n* = 6 slices from 3 mice per group). Scale bar, 40 μm. **c**, **d** Confocal images (**c**) and quantification (**d**) of the co-expression of G6PD and neuronal marker NeuN in the cortex of 2.5-month-old WT and 5×FAD mice (*n* = 6 slices from 3 mice per group). Scale bar, 40 μm. Data represent mean ± SEM. For all statistical analyses, statistical significance was analyzed using two-tailed unpaired Student's t-test.


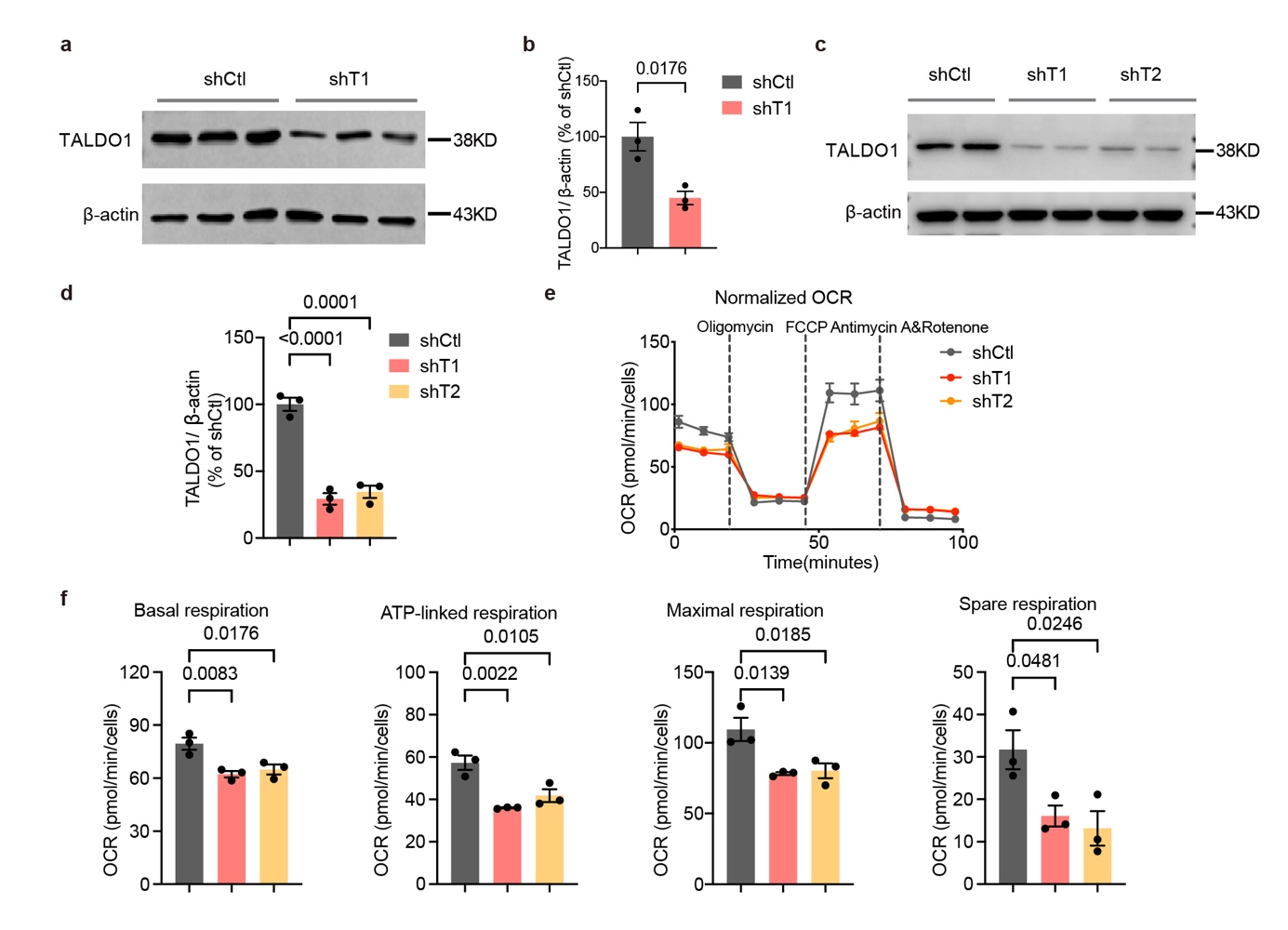


**Fig. S4 Neuronal *Taldo1* knockdown reduces glucose metabolism and disrupts metabolic homeostasis.**

**a,** **b** Western blot analysis (**a**) and quantification (**b**) of TALDO1 expression in primary neurons with *Taldo1* knockdown (*n* = 3 indepent replicates). **c**, **d** Protein expression (**c**) and quantification (**d**) of TALDO1 in HT22 cells knocked down with 2 sequences of shRNAs (shT1 and shT2) (*n* = 3 indepent replicates). **e** OCR measured in mitochondria of HT22 cells (*n* = 3 indepent replicates). **f** Quantification of mitochondrial basal respiration, ATP production, oxidative phosphorylation rate, and respiratory potential based on OCR (*n* = 3 indepent replicates). Data represent mean ± SEM. For **b**, statistical significance was analyzed using two-tailed unpaired Student's t-test. For **d** and **f**, statistical significance was analyzed using two-way ANOVA followed by Dunnett’s post hoc test.


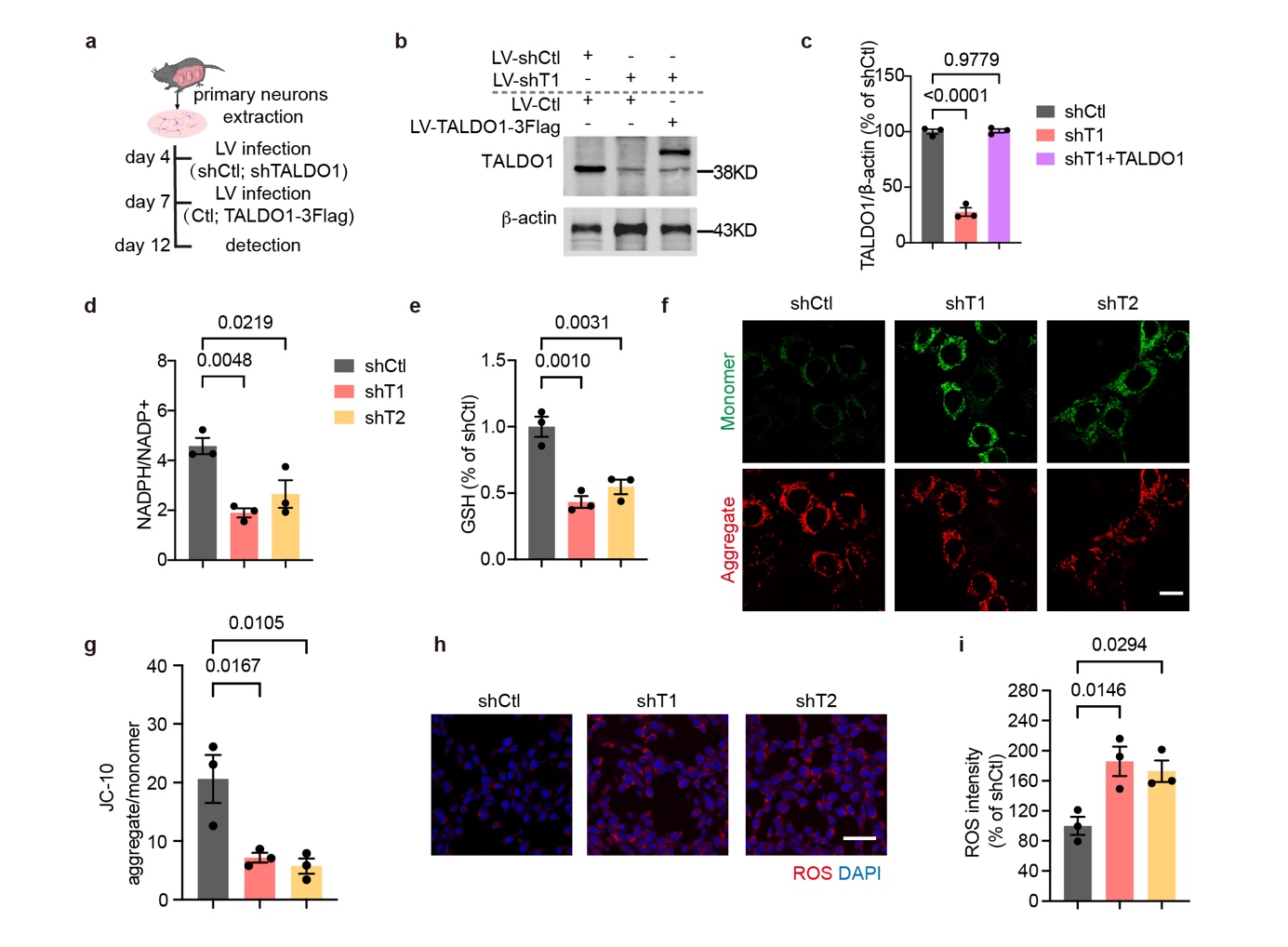


#### Fig. S5 Knockdown of *Taldo1* causes oxidative stress and mitochondrial dysfunction.

**a** Schematic diagram showing the isolation and culture process of primary neurons from C57BL/6J mouse embryos. **b**, **c** Western blot analysis (**b**) and quantification (**c**) of TALDO1 expression in primary neurons with *Taldo1* knockdown or with *Taldo1* overexpression after knockdown (*n* = 3 indepent replicates). **d**, **e** NADPH/NADP^+^ ratio (**d**) and GSH content (**e**) in HT22 cells (*n* = 3 indepent replicates). **i**, **j** Mitochondrial membrane potential measured by JC-10 staining (**i**) and quantification of JC-10 aggregate/monomer ratio (**j**) in HT22 cells (*n* = 3 slices with 6 views in each slice analyzed). Scale bar, 40 μm. **k**, **l** Confocal images (**k**) and quantification (**l**) of ROS content in HT22 cells (*n* = 3 slices with 6 views in each slice analyzed). Scale bar, 40 μm. Data represent mean ± SEM. For all statistical analyses, statistical significance was analyzed using one-way ANOVA followed by Dunnett’s post hoc test.

####
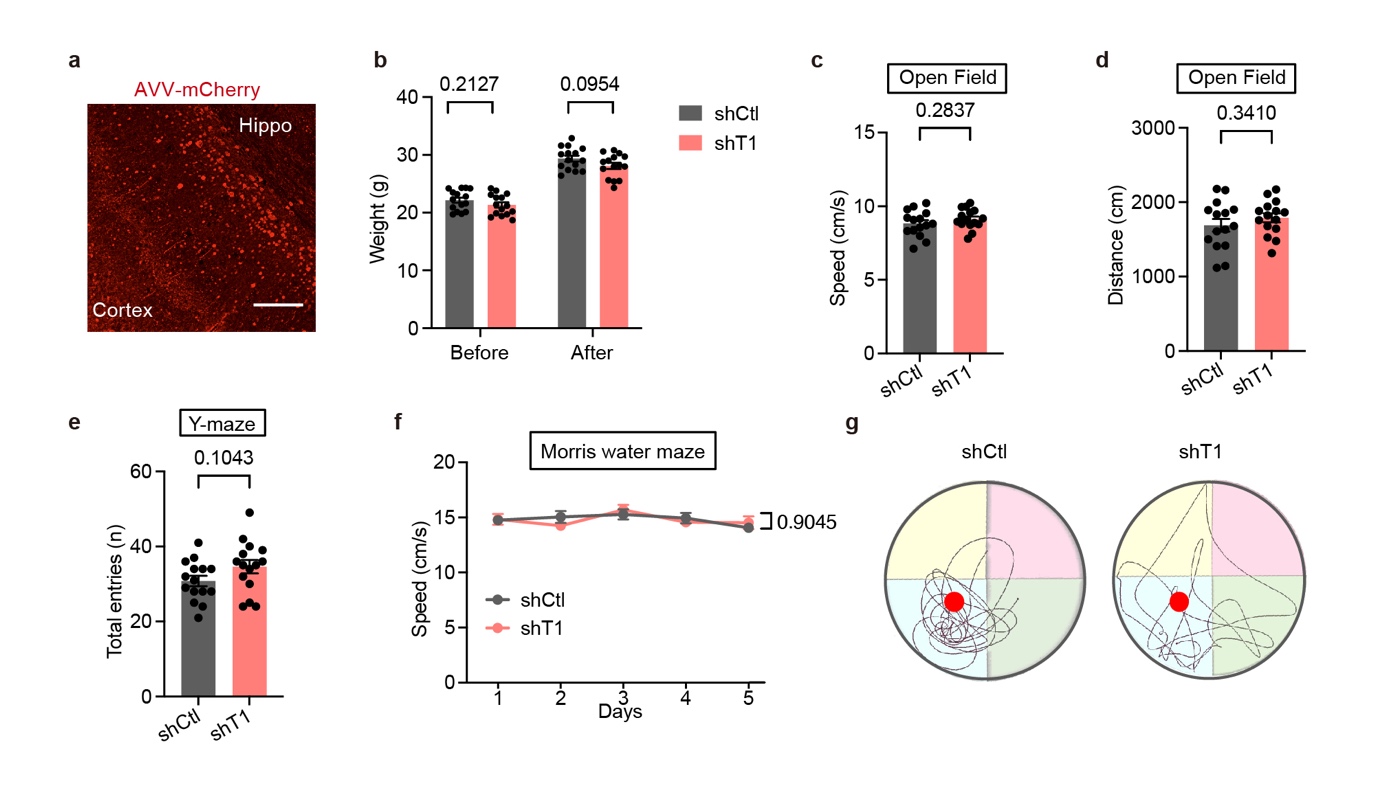
Fig. S6 Neuronal knockdown of *Taldo1* in WT mice does not affect body weight and locomotor activity.

#### a Confocal image showing the expression of mCherry in the hippocampus and cortex. Scale bar, 40μm. b Changes in body weight before and 8 weeks after virus injection. c, d Average moving speed (c) and total movement distance (d) in open field test. e Total entries into arms in Y maze. f Average swimming speed of mice during the training period of MWM. g Representative swimming trajectories of mice during the probe trial of MWM. The red circle represented the hidden platform. (*n* = 15). Data represent mean ± SEM. For b-e, statistical significance was analyzed using two-tailed unpaired Student's t-test. For f, statistical significance was analyzed using two-way followed by ANOVA Fisher’s LSD post hoc test.


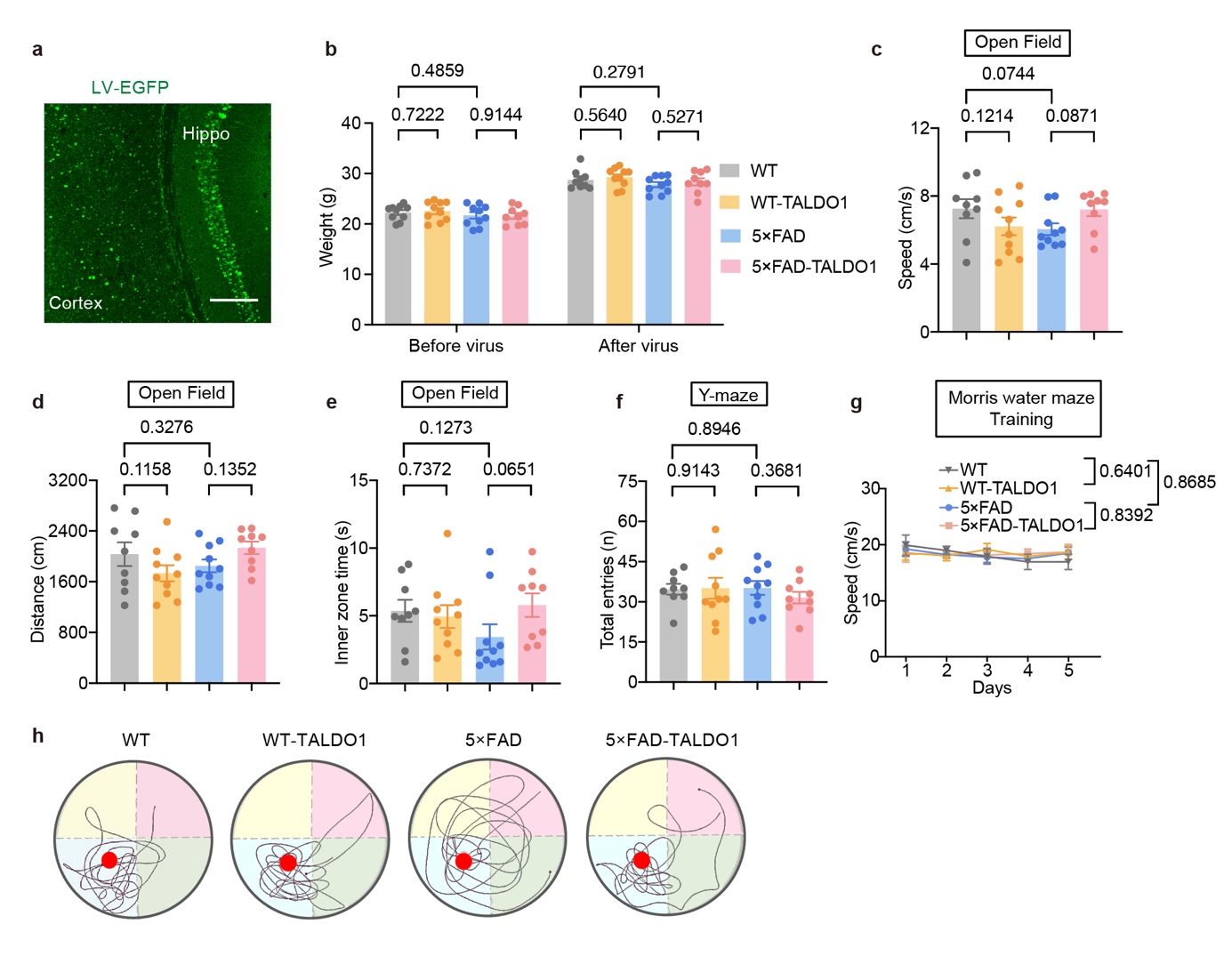


#### Fig. S7 Effects of restoring neuronal expression of TALDO1 on body weight, locomotion, anxiety behavior and neuronal skeleton in 5×FAD mice.

**a** Confocal image showing the expression of EGFP in the hippocampus and cortex. Scale bar, 40μm. **b** Body weight of WT and 5×FAD mice before and after 1.5 months lentiviral injection. **c**-**e** Average moving speed (**c**), total movement distance (**d**), and time spent in the central area (**e**) in open field test. **f** Total number of entries of arms in Y maze test. **g** Average swimming speed during the training period of MWM. **h** and representative swimming trajectories during the probe trial in the MWM. The red circle represented the hidden platform. (*n* = 9 for WT and 5×FAD-TALDO1, *n* = 10 for other two groups in open field and Y maze; *n* = 8 for WT, *n* = 10 for WT-TALDO1, *n* = 9 for 5×FAD and 5×FAD-TALDO1 in MWM). Data represent mean ± SEM. Statistical significance was analyzed using two-way ANOVA followed by Fisher’s LSD post hoc test.
