# Supplementary figures and images for: Transaldolase 1 contributes to pentose phosphate pathway disruption and synaptic dysfunction in Alzheimer’s disease
Source: Transl Neurodegener. 2026 Jul 29;15:35. doi: 10.1186/s40035-026-00567-z (PMC13418138; doi:10.1186/s40035-026-00567-z)

Fig 2a

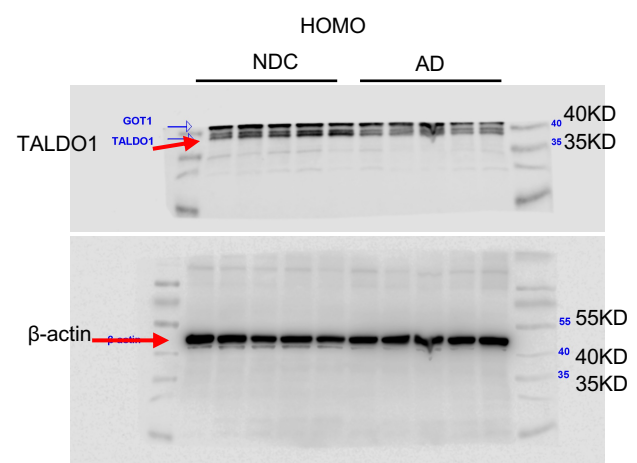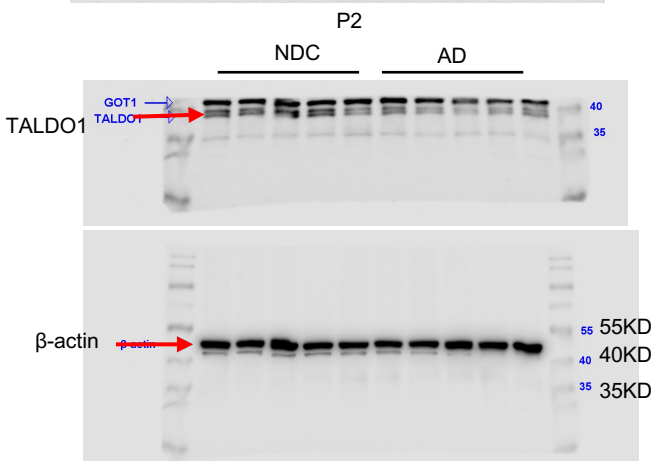

Fig 2d

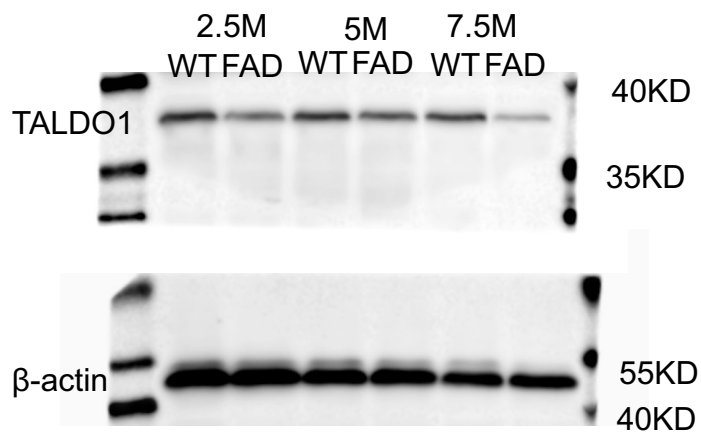

Fig 4g

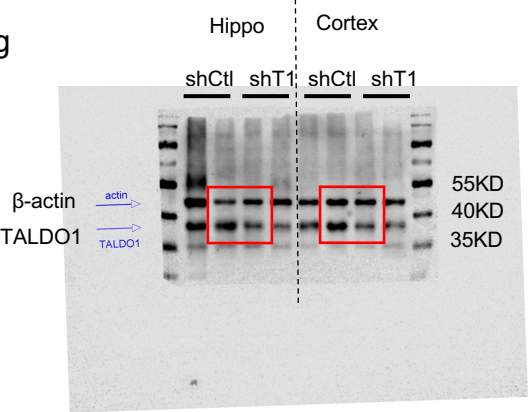

Fig 4m

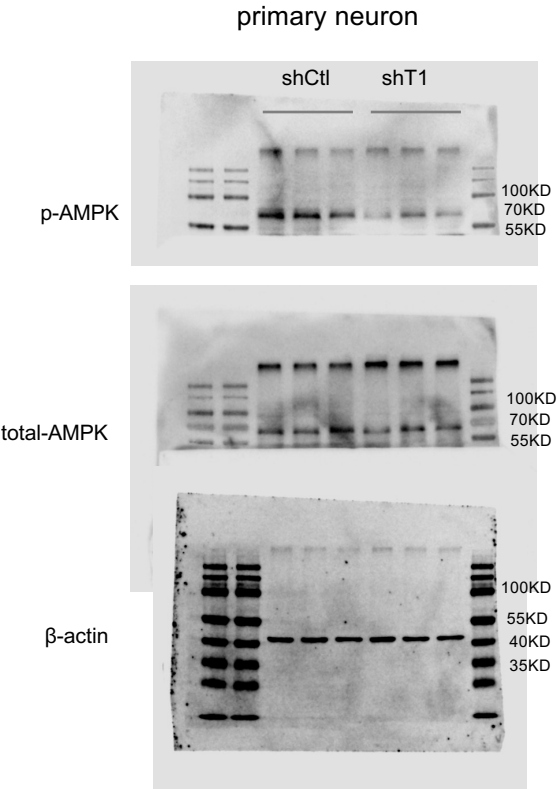

Fig 4o

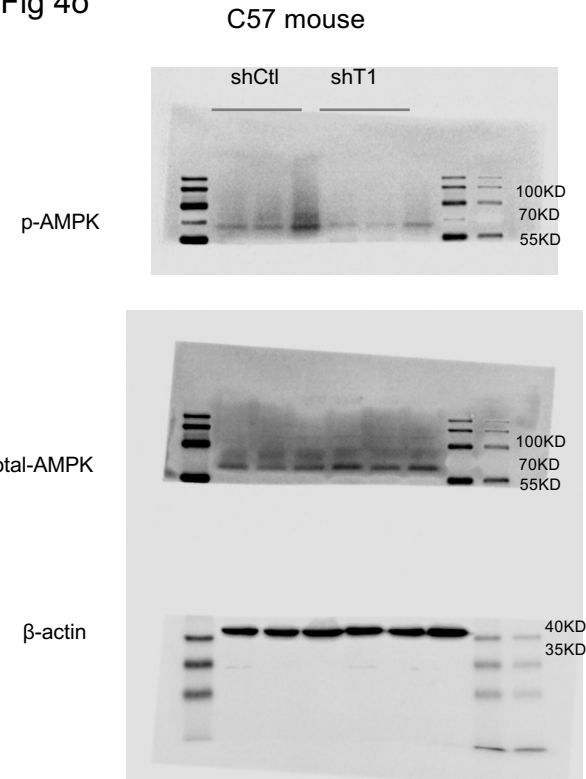

Fig 7a

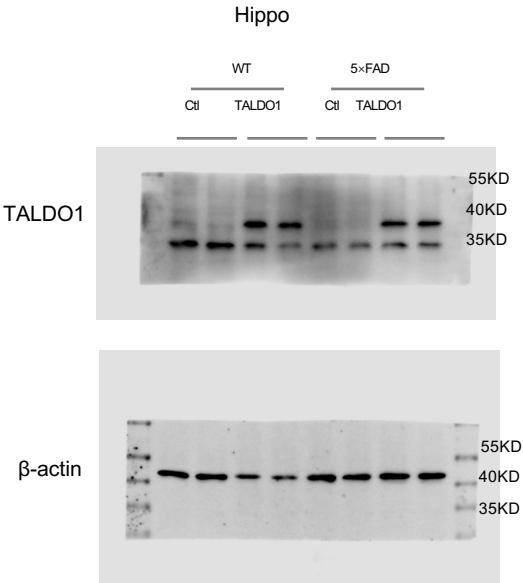

Fig 7c

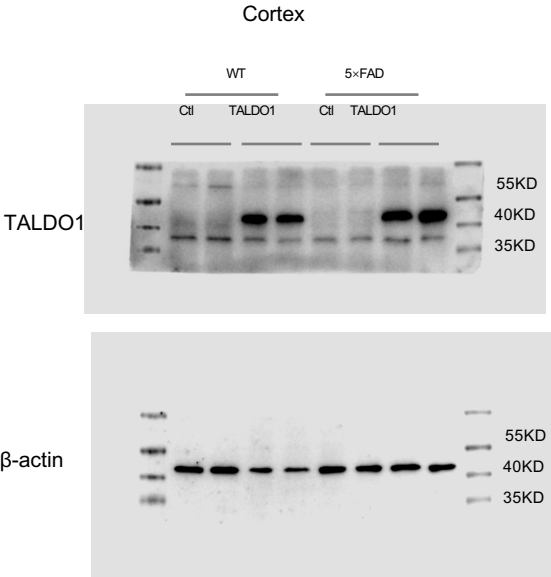

Fig 7o

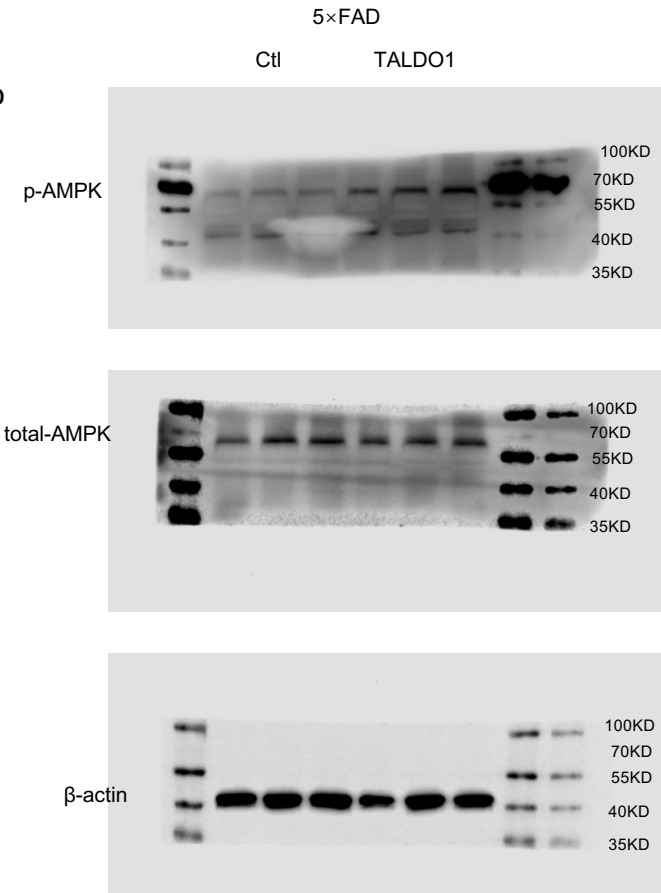

Sup2d

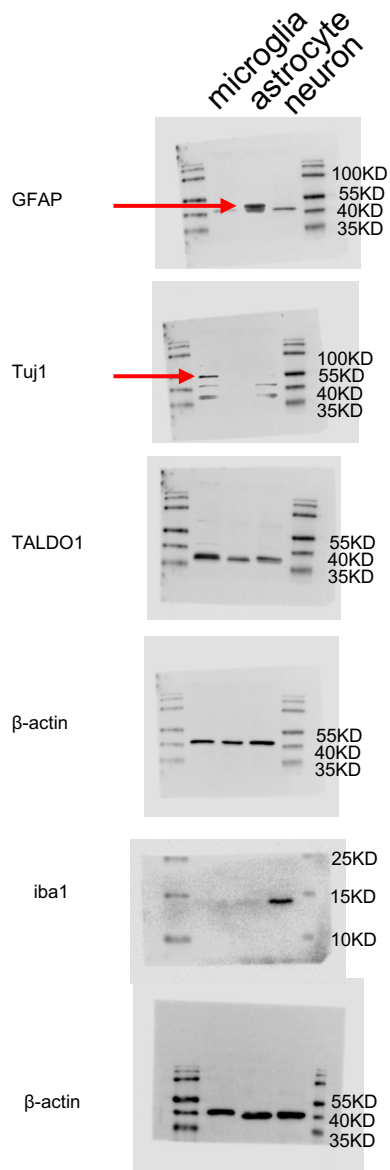

Sup2h

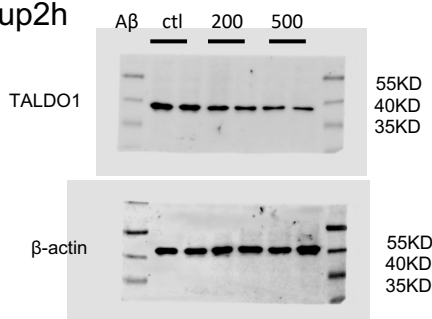

Sup2k

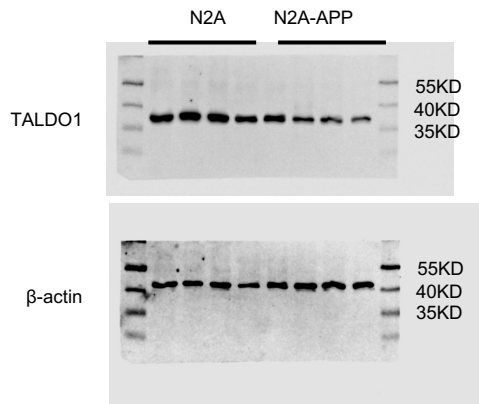

Sup4a

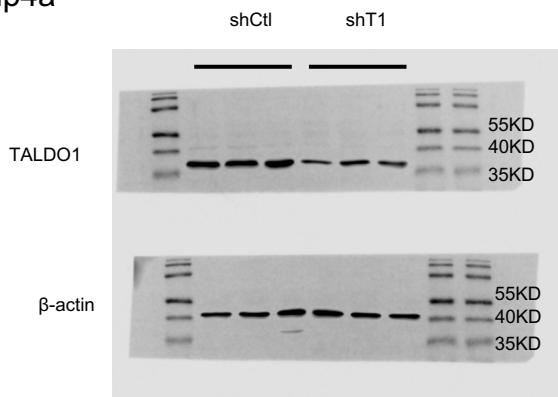

Sup4c

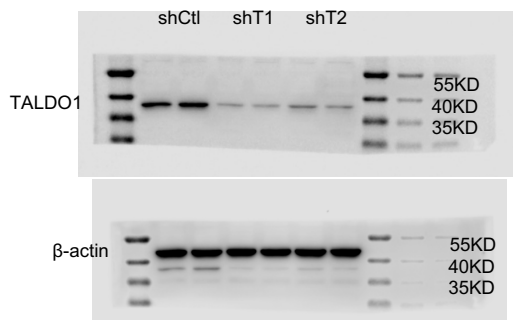

Sup5b

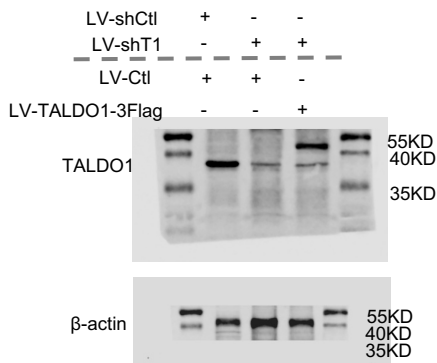

Supplement: Supplementary file 5 — Additional file 5 Unprocessed western blots. [file 40035_2026_567_MOESM5_ESM.pdf]
